# *Trappc9* deficiency causes parent-of-origin dependent microcephaly and obesity

**DOI:** 10.1371/journal.pgen.1008916

**Published:** 2020-09-02

**Authors:** Zhengzheng S. Liang, Irene Cimino, Binnaz Yalcin, Narayanan Raghupathy, Valerie E. Vancollie, Ximena Ibarra-Soria, Helen V. Firth, Debra Rimmington, I. Sadaf Farooqi, Christopher J. Lelliott, Steven C. Munger, Stephen O’Rahilly, Anne C. Ferguson-Smith, Anthony P. Coll, Darren W. Logan

**Affiliations:** 1 Wellcome Sanger Institute, Wellcome Genome Campus, Cambridge, United Kingdom; 2 MRC Metabolic Diseases Unit, Wellcome Trust-Medical Research Council Institute of Metabolic Science, University of Cambridge, Cambridge, United Kingdom; 3 Institut de Génétique et de Biologie Moléculaire et Cellulaire, Centre National de la Recherche Scientifique, Institut National de la Santé et de la Recherche Médicale, Université de Strasbourg, France; 4 The Jackson Laboratory, Bar Harbor, Maine, United States of America; 5 Cancer Research UK Cambridge Institute, University of Cambridge, Cambridge, United Kingdom; 6 Department of Clinical Genetics, Addenbrooke’s Hospital, Cambridge, United Kingdom; 7 University of Cambridge Metabolic Research Laboratories and NIHR Cambridge Biomedical Research Centre, Addenbrooke's Hospital, Cambridge, United Kingdom; 8 Department of Genetics, University of Cambridge, Cambridge, United Kingdom; The Babraham Institute, UNITED KINGDOM

## Abstract

Some imprinted genes exhibit parental origin specific expression bias rather than being transcribed exclusively from one copy. The physiological relevance of this remains poorly understood. In an analysis of brain-specific allele-biased expression, we identified that *Trappc9*, a cellular trafficking factor, was expressed predominantly (~70%) from the maternally inherited allele. Loss-of-function mutations in human TRAPPC9 cause a rare neurodevelopmental syndrome characterized by microcephaly and obesity. By studying *Trappc9* null mice we discovered that homozygous mutant mice showed a reduction in brain size, exploratory activity and social memory, as well as a marked increase in body weight. A role for *Trappc9* in energy balance was further supported by increased *ad libitum* food intake in a child with TRAPPC9 deficiency. Strikingly, heterozygous mice lacking the maternal allele (70% reduced expression) had pathology similar to homozygous mutants, whereas mice lacking the paternal allele (30% reduction) were phenotypically normal. Taken together, we conclude that *Trappc9* deficient mice recapitulate key pathological features of *TRAPPC9* mutations in humans and identify a role for *Trappc9* and its imprinting in controlling brain development and metabolism.

## Author summary

Every person has inherited two copies of each gene, one from each parent. In most cases two copies contribute equally. Other genes may express only from one parental copy, or express both copies but prefer one over another, a phenomenon called genomic imprinting. Here we first investigated the expression bias between two parental copies in the brain, then focused on one gene with maternal biased expression called *Trappc9*. Mutations in human TRAPPC9 cause a neurodevelopmental disorder marked by smaller brain size and increased body weight. Indeed, we found mice lacking of *Trappc9* showed a reduction in brain size, behavioral changes and a marked increase in body weight. A functional role for *Trappc9* in metabolism was further supported by increased food intake in a child lacking this gene. Interestingly, we also found mice lacking the preferred copy (from mother) of the gene showed similar disorders, whereas mice lacking the other copy (from father) appeared normal. We provide an animal model of a rare genetic disease and identify a role for *Trappc9* and its imprinting in controlling brain development and metabolism.

## Introduction

Allelic imbalance, the unequal level of expression between the two alleles of a gene, has been extensively reported in the mammalian genome [1–3]. Imprinted genes exhibiting clear parent-of-origin effects have been well-characterized [4]. These canonical imprinted genes are predominantly expressed from one of the two parental alleles and tightly regulated to control gene dosage [3]. They exhibit diverse functions in pre- and post- natal growth, often with roles in energy homeostasis and behavior [5, 6].

Imprinted genes can exhibit a complex pattern of tissue-specific parental-specific expression, leading to unique physiological consequences. For instance, the paternally inherited copy of the imprinted *growth factor receptor bound protein 10* (*Grb10*) is exclusively expressed in the murine brain and spinal cord, whereas the maternally inherited copy is expressed in the rest of the body [7]. As a result, paternal heterozygous mutant mice have abnormal social hierarchies [8], whereas mice deficient in the maternally inherited copy exhibit increased body weight [7]. In human, *GRB10* is also paternally expressed in the brain, but biallelically expressed in most non-neuronal tissues except placental villous trophoblasts and skeletal muscle (maternally expressed) [9, 10]. Disruption of *GRB10* imprinting is suggested to cause Russell-Silver syndrome (RSS), characterized by pre- and post- natal growth retardation and dysmorphology [11].

Imprinted genes are abundant in the brain [5, 12, 13] and abnormal imprinting can cause cognitive developmental disorders [5]. In humans, the loss of maternally expressed Ubiquitin protein ligase E3A (*UBE3A*) is a cause of Angelman Syndrome (AS), a neurodevelopmental disorder [14]. In mice, depletion of maternally expressed *Ube3a* results in many deficits including disruption of circadian rhythm [15], impaired synaptic plasticity and learning performance [16, 17]. *Ube3a* and *UBE3A* are predominantly expressed from the maternally inherited copy in the brain and equally expressed from both copies outside the nervous system [12, 18].

High-throughput sequencing studies have recently identified various forms of “non-canonical” allelic imbalance, one of which encompasses parent-of-origin specific expression bias rather than being transcribed exclusively from one parental copy [19]. Such effects have been described in particular regions of the brain [12, 13, 20, 21]. However, the functional significance and phenotypic consequences of this new type of imprinting remain largely uncharacterized.

*Trappc9* gene (human synonym: NIBP) encodes a protein that forms a component of mammalian TRAPP (TRAnsport Protein Particle) complex involved in vesicular protein trafficking between the endoplasmic reticulum (ER) and Golgi apparatus, and has also been implicated in NF-κB signaling [22, 23]. *Trappc9* is reported to be highly expressed within the murine brain, with transcripts abundant in the hippocampus and hypothalamus as well as the glomeruli and mitral layers of the olfactory bulb [24]—regions with a role in the control of metabolism, memory and behavior. In humans, both homozygous and compound heterozygous mutations in *TRAPPC9 (NIBP*) associate with developmental delay, microcephaly (95% reported cases), and obesity (52% reported cases) [25–31]. Reports to date have provided only limited insights into the impact of TRAPPC9 deficiency on energy balance and body composition, although obesity is frequently noted. Interestingly, human *TRAPPC9* and murine *Trappc9* both reside within a cluster of imprinted genes, known as the *PEG13-KNCK9* cluster [32], suggesting its expression might be biased towards one of the parental alleles.

In this study, we quantified the allele-specific transcriptome exclusive to murine brain and non-brain structures of the olfactory system. We identified *Trappc9* as a gene exhibiting reproducible brain-specific, parental-biased expression, with 30% of the transcripts derived from the paternal allele and 70% from the maternally inherited chromosome. To examine the role of *Trappc9* allelic biased expression we analyzed *Trappc9* deficient mice. These mice phenocopy the major features of human TRAPPC9 deficiency syndrome with a reduction in brain size, altered behavior and memory defect, as well as a marked increase in body weight and fat mass. Strikingly and consistent with its imprinting, heterozygous mice lacking the maternal allele (*Trappc9* ^m(tm1a)/p+^, 70% reduced expression) have a phenotype similar to homozygous mutant mice, whereas mice lacking the paternal allele are phenotypically normal. Our findings indicate that the brain-specific, parental-biased allelic expression of *Trappc9* regulates brain size, behavior and body weight.

## Results

### Allelic imbalance is robust in the olfactory system

To reliably determine allele-biased gene expression in neuronal versus non-neuronal structures, we analyzed genome-wide allelic expression in the olfactory bulb (OB) and main olfactory epithelium (MOE). The OB and MOE are important sequential relays in the sensory circuitry that promote learned and innate olfactory-mediated behavior in mice, specifically social behavior [33]. Whereas the OB is of neuronal origin and part of the brain, the MOE resides in the nasal cavity and is placodal in origin [34]. Comparing allele-specific expression in the OB and MOE, we aimed to understand the tissue-specific role of allelic imbalance in developmentally distinct but functionally related anatomical regions.

To measure allele-specific gene expression we used strain-specific single-nucleotide polymorphisms (SNPs) and indels present in the genome of distantly related inbred mouse strains C57BL/6J (B6) and CAST EiJ (CAST). B6 and CAST hybrid transcriptomes were generated using transcripts containing strain-specific genetic variations (S1 Fig) [20, 35, 36]. We aligned reads to the diploid transcriptome with Bowtie and used EMASE [37] to quantify allele-level expression. Parent-of-origin specific gene expression was only considered for transcripts with ≥5 unique reads (SNP-containing and resolved) in both reciprocal crosses. Our analysis (S2, S3 and S4 Figs) resulted in reliable quantification of allele-specific expression of 12,101 genes in OB and 11,418 genes in MOE, representing approximately 50% of the total expressed genes (24,152 in OB and 22,952 in MOE) in the respective tissues.

**Fig 1:**
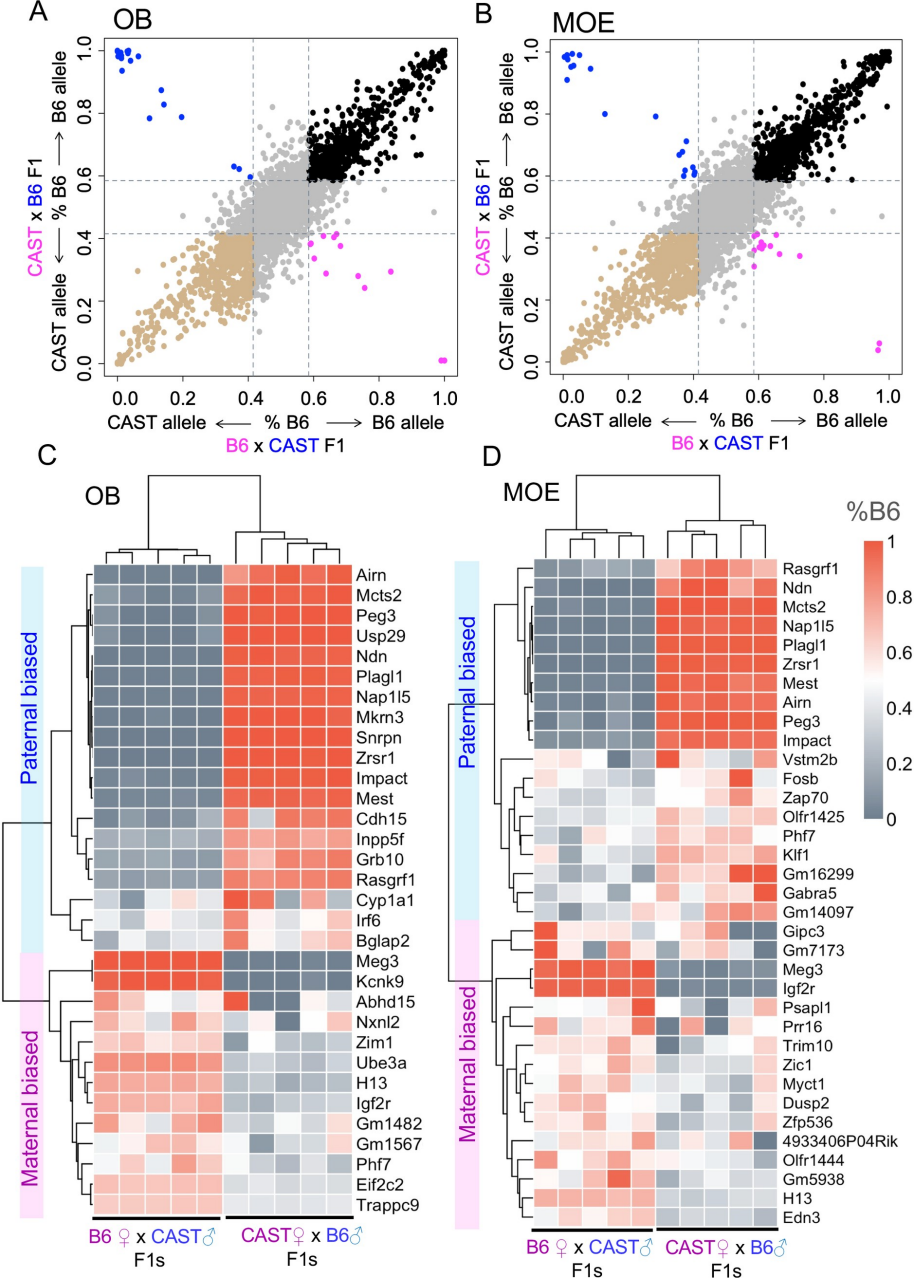
Analysis of allelic imbalance in the mouse olfactory system. **(A-B)** Scatter plots of the average allelic bias ratios in olfactory bulb (OB) and main olfactory epithelium (MOE) in F1 individuals (females, aged 14 weeks) from reciprocal crosses between C57BL/6J (B6) and CAST/EiJ (CAST) strains. Pink: maternal biased expression; blue: paternal biased expression. Black: B6 strain biased expression; brown: CAST strain biased expression. The cut off ratio applied for biased expression is 0.6:0.4. (**C-D)** Heatmaps of parental allelic ratios for individual genes in each sample. The allelic ratios are quantified as the ratio of B6 allelic expression: %B6 = B6/(B6+CAST) and are clustered by samples (each column) and by allelic ratio of the genes expressed in OB and MOE (each row). Genes with average %B6 ratios >0.6 in B6 ♀x CAST ♂F1 mice and ratios <0.4 in CAST♀x B6 ♂F1 mice, are maternally-biased; whereas genes with average %B6 ratios <0.4 in B6♀x CAST♂F1 mice and %B6 ratios >0.6 for CAST♀xB6♂F1 mice are paternally-biased.

As expected [2, 38], we found large strain effects on allelic expression (Fig 1A and 1B, S1 Data). Strain-specific differences in gene expression were present in 15% of expressed genes in the OB, and 18% in the MOE (defined by a 0.6:0.4 ratio or larger between B6 and CAST, observed in both reciprocal crosses). Similar numbers of genes were biased to the B6 strain versus biased to the CAST strain. Most notably, strain-biased expression of olfactory receptors was enriched in the main olfactory epithelium (*χ*^*2*^ test, *p*<0.0001), with 184 olfactory receptor genes, or 41% of the total detectable olfactory receptors, expressed at unequal levels depending on the strain-of-origin (S1 Table).

**Fig 2:**
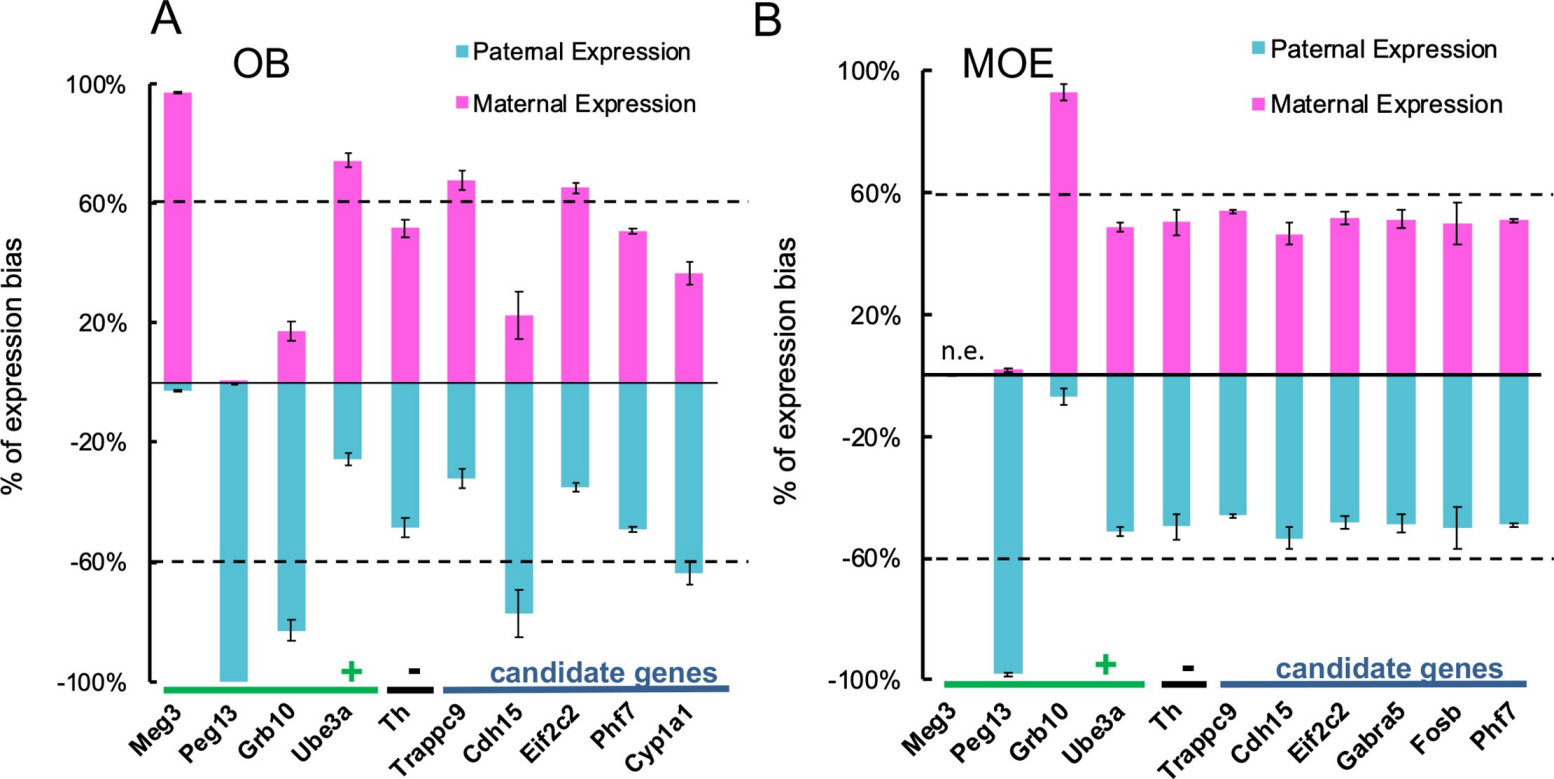
Validation of parental allelic biased gene expression. Allele-specific gene expression was determined for selected genes using Allelic Discriminant qRT-PCR on F1 reciprocal hybrid samples (females, aged 14 weeks) of the (**A)** olfactory bulb and **(B)** main olfactory epithelium. The percentage of parental and maternal expression is shown as an average of the results from two reciprocal crosses with n = 5 for each cross. Four genes with previous identified parent-of-origin expression (*Meg3*, *Peg13*, *Ube3a and Grb10*) were as positive controls and *Th* was a negative control. As expected, *Meg3* showed monoallelic maternal expression and *Peg13* showed monoallelic paternal expression; *Meg3* did not express in non-brain tissue while *Peg13* did. *Ube3a* showed brain-specific imprinting with maternal allelic-biased expression. *Grb10* shows opposite imprinting in the brain (maternal allelic-biased) versus in a non-brain tissue (paternal allelic-biased). *Th* expression was bi-allelic in both tissues as expected. For candidate genes, we confirmed that *Trappc9* and *Eif2c2(Ago2)* showed maternal allelic-biased and *Cdh15* showed paternal allelic-biased expression, both were in OB but not in MOE, suggesting brain specific imprinting. The expression of *Garba5* and *Fosb* in MOE and *Phf7* in both tissues was bi-allelic; but *Cyp1a1* showed a paternal biased expression in the OB, as a previously unknown gene with brain-specific imprinting.

We quantified 54 genes with robust parent-of-origin biased expression (defined by a 0.6:0.4 ratio or larger between maternal and paternal allelic expression [39], observed in both reciprocal crosses) - 32 genes in OB and 35 genes in MOE with 13 genes showing parent-of-origin allelic biased expression in both tissues (Fig 1C and 1D, S2 Data). This accounted for ~0.3% of total genes with allele-specific quantified expression in each tissue. Of the genes identified, ~75% in OB and ~40% in MOE were previously defined to be imprinted (S5 Fig) [39, 40]. To validate our dataset, we used allelic discriminative quantitative-PCR to determine allelic expression of twelve genes (Fig 2), including four positive controls (*Meg3*, *Peg13*, *Grb10*, *Ube3a*), one negative control (*Th*) and seven candidate genes identified in this study. Of the seven candidates, three have not been reported previously (*Cyp1a1*, *Fosb*, *Phf7*), whereas four show conflicting data in previous studies (*Trappc9*, *Eif2c2 (Ago2)*, *Cdh15*, *Gabar5*) [12, 21, 40, 41].

Consistent with previous studies, *Meg3* showed mono-allelic expression of the maternally-inherited allele in the OB and did not express in the MOE, whereas *Peg13* showed mono-allelic expression of the parental allele in both the OB and MOE. *Grb10* showed reciprocal allelic expression in OB (parental) and MOE (maternal) and *Ube3a* showed brain-specific imprinting in the OB but not MOE; whereas *Th* showed equal expression from both parental alleles in both the OB and MOE as expected (Fig 2). Of our candidate genes, *Cdh15* and *Cyp1a1* showed ~80% and ~65% paternal-biased expression in the OB, respectively, whereas *Trappc9* and *Eif2c2* (*Ago2*) showed ~70% and ~65% maternal biased expression in the OB, with expression of all four genes being bi-allelic (~50:50 paternal: maternal) in the MOE. We were unable to validate parental-biased expression of *Garab5* or *Fosb* in the MOE and *Phf7* expression in either tissue (Fig 2, S3 Data).

Our analyses, therefore, identified allelic biased expression within the OB and MOE of the mouse olfactory system, and validated parent-of-origin dependent allelic-biased expression of four candidate genes: *Trappc9*, *Cyp1a1*, *Eif2c2 (Ago2) and Cdh15* within the OB. We next focused on understanding the biological relevance of this brain-specific imprinting, using one of these candidate genes, the *Trafficking protein particle complex 9* (*Trappc9)*.

### Characterization of allelic imbalance in Trappc9 mutant mice

**Fig 3:**
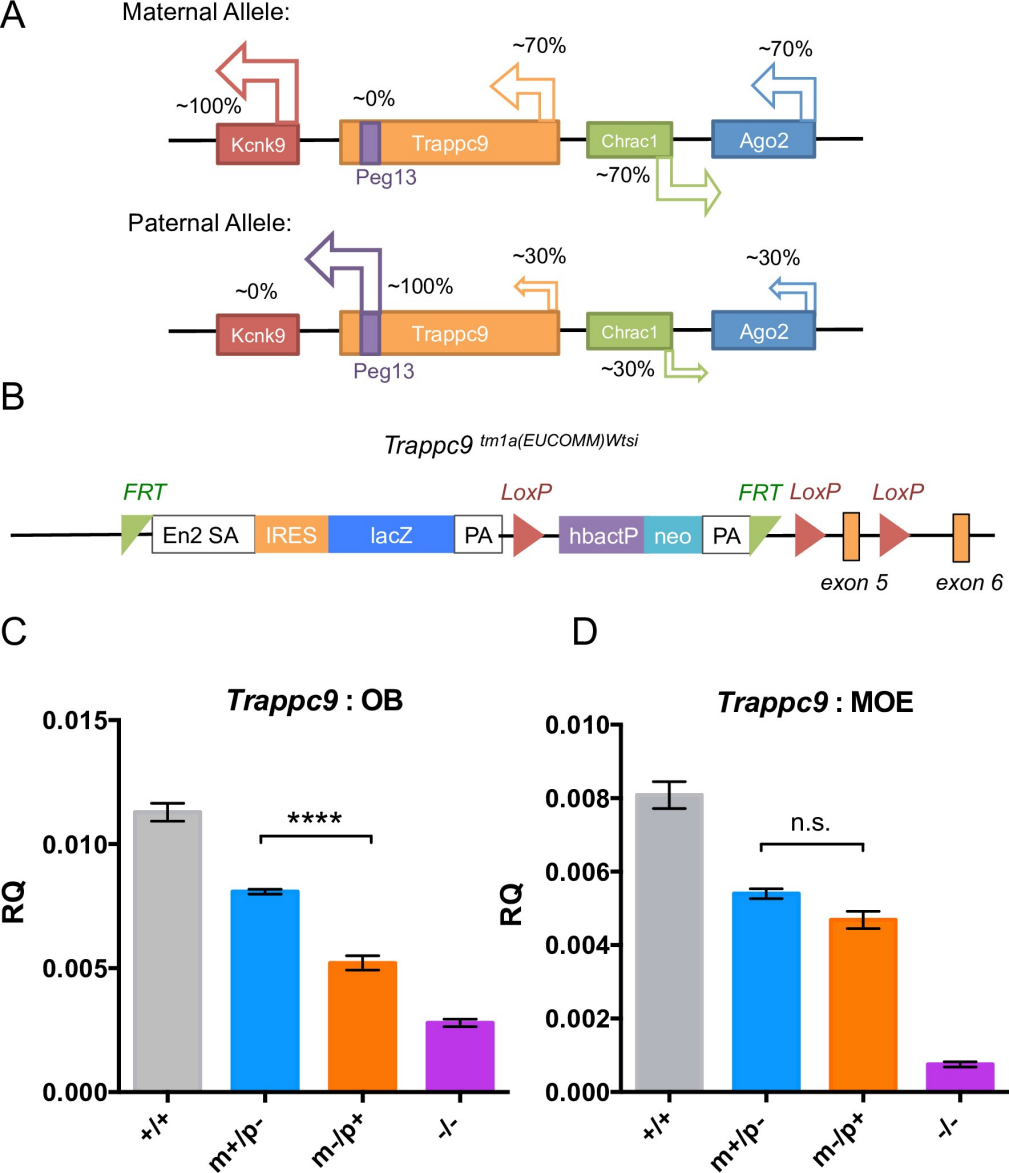
Generation of *Trappc9* deficient mice. (**A)** Schematic depiction of the *Trappc9* locus and *Peg13* imprinted cluster on mouse chromosome 15 with allelic biased expression indicated. (**B)** Schematic view of the *tm1a(EUCOMM)*^*wtsi*^ conditional gene-trap cassette in intron 4 of *Trappc9*. (**C-D)** Confirmation of maternal-biased *Trappc9* expression in heterozygous *Trappc9* knock-out mice (*p<0.05, ****p<0.0001, one-way ANOVA with Dunnet’s *post hoc* test). *Trappc9* mRNA abundance was determined relative to β-actin in OB (**C)** and MOE (**D)** of wild-type (+/+), maternal (m-/p+) / paternal (m+/p-) heterozygous and homozygous (-/-) *Trappc9* deficient mice using quantitate RT-PCR (males, n = 5 per genotype).

Consistent with a role in brain function, mouse *Trappc9* was highly expressed in the postnatal brain (S6 Fig). Our imprinting study suggested mouse *Trappc9* was expressed 70% from the maternal allele with 30% from paternal allele, specifically in the brain (OB) with equal allelic expression in other tissues (MOE). This observation has been confirmed in previous studies of allelic biased expression [12, 35]. Although it is currently unclear whether human *TRAPPC9* is imprinted, both human and mouse *TRAPPC9/Trappc9* reside in the characterised *Peg13-Kcnk9* imprinting cluster [32]. Allelic biased expression of this cluster, including the maternally-expressed brain-specific potassium channel *KCNK*/*Kcnk9*, is thought to be regulated by the non-coding RNA *PEG13/Peg13* expressed from an intron within the *TRAPPC9/Trappc9* gene (Fig 3A) [12, 32, 42].

To study the role of Trappc9 in brain function and disease and to understand the biological importance of its imprinting, we generated *Trappc9* knock-out mice (*Trappc9* ^*tm1a[EUCOMM)Wtsi*^) of four genotypes: 1) homozygous *Trappc9* knockouts (*Trappc9* ^-/-^); 2) heterozygous *Trappc9* knockout mice lacking expression from the paternal allele (*Trappc9* ^*m+/p-*^); 3) the maternal allele (*Trappc9* ^m-/p+^) and 4) wild type mice (*Trappc9* ^+/+^)(Fig 3B) [43]. Consistent with our imprinting study, heterozygous paternal (*Trappc9* ^*m+/p-*^), maternal (*Trappc9* ^m-/p+^) and homozygous (*Trappc9* ^-/-^) knock-outs showed respectively 25%, 60% and 80% reduction in *Trappc9* transcript abundance in the OB (Fig 3C, *p*<0.0001, one-way ANOVA with Dunnett’s *post hoc* test, S4 Data). A similar reduction was confirmed in other brain regions by RNA sequencing. Thus, in the hippocampus, the *Trappc9* transcript levels in paternal heterozygous, maternal heterozygous and homozygous mutant mice decreased by respectively 18%, 53% and 74%, of the levels seen in wild-type mice (S7A and S7C Fig). Similarly, in the hypothalamus, transcript levels in the same three mutant genotypes showed 23%, 63% and 79% reduction compared to the wild-type levels (S7B and S7D Fig). Heterozygous loss of *Trappc9* thus results in a parent-of-origin dependent decrease in *Trappc9* transcript abundance in the brain. In contrast, in the MOE *Trappc9* expression levels decreased by respectively 39%, 43% and 83% of wild-type levels, in the paternal, maternal and homozygous knock-outs respectively (Fig 3D, *p*>0.05, S4 Data), reflecting a lack of *Trappc9* imprinting in non-neuronal tissue. *Trappc9* transcripts remaining in the homozygous *Trappc9* knock-out (21~25%, Fig 3C) likely reflected alternative splicing and do not result in protein expression, since Trappc9 protein expression was fully lost in the homozygous knock-out (S8 Fig).

A parent-of-origin dependent decrease in *Trappc9* was also evident at the protein level, whereby loss of the maternal allele (*Trappc9* ^*m-/p+*^) resulted in a greater loss of Trappc9 protein expression compared to loss of the paternal allele (*Trappc9* ^*m+/p-*^) in the OB, but not MOE (S8 Fig). Importantly, the *Trappc9* gene-trap did not affect expression of neighboring imprinted genes, including *Kcnk9* and the non-coding RNA *Peg13* (S9A and S9B Fig). Of the upstream genes *Chrac1* was modestly increased in expression only in the OB of *Trappc9* ^*m-/p+*^ mice but unchanged in *Trappc9* ^*m+/p-*^ and *Trappc9* ^*-/-*^ mice, while *Eif2c2(Ago2)* RNA abundance showed an increase in *Trappc9* ^*-/-*^ mice but remained unchanged in *Trappc9* heterozygous knockouts (S9C and S9D Fig). Indeed, loss of *Trappc9* had limited impact on the transcriptome of the hippocampus and hypothalamus (S7C and S7D Fig).

### *Trappc9* deficient mice develop microcephaly in a parent-of-origin dependent manner

**Fig 4:**
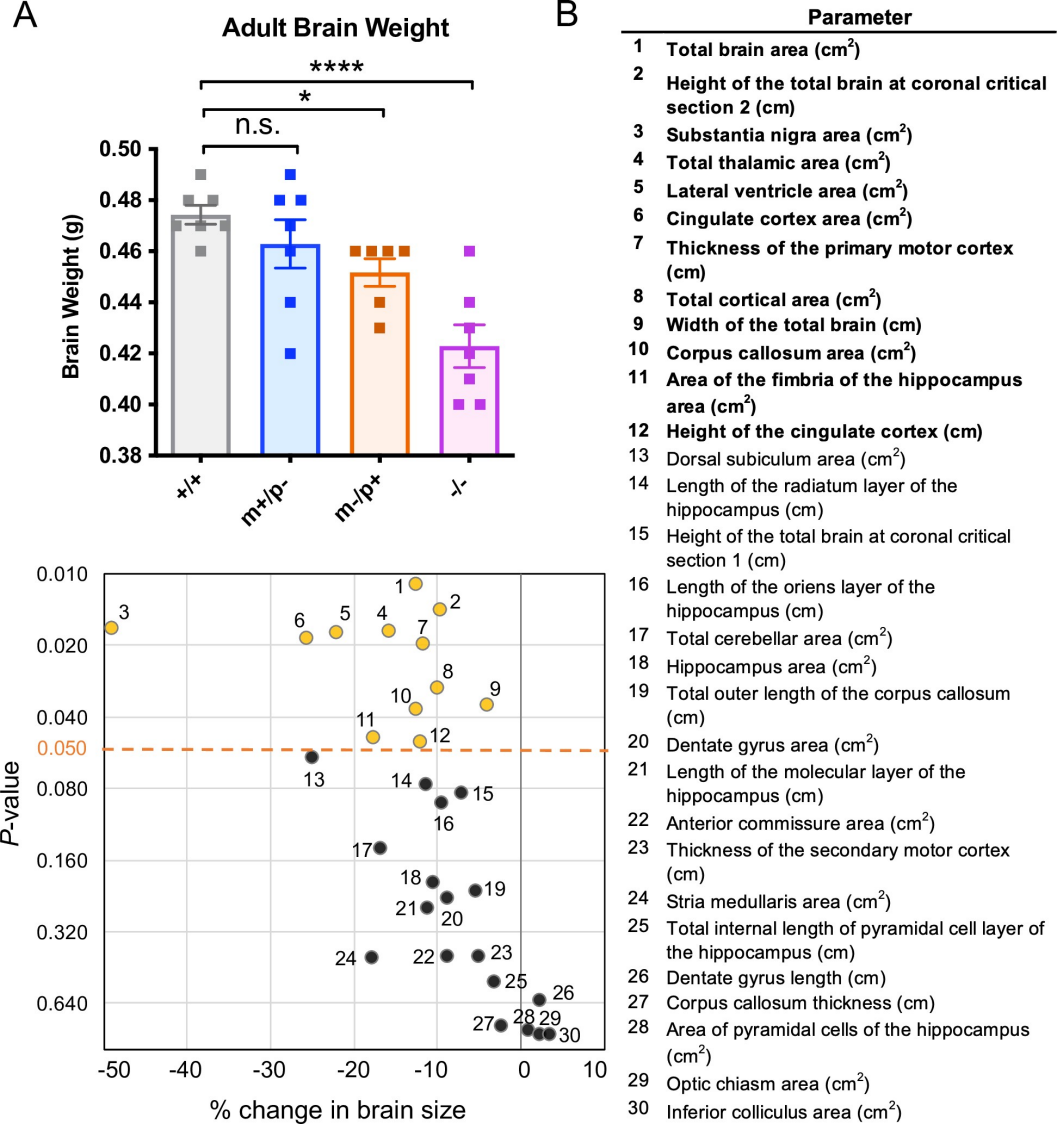
*Trappc9* deficient mice show a decrease in brain weight and size. **(A)** *Trappc9* deficient mice show a decrease in brain weight in a parent-of-origin manner. *Trappc9* m-/p- mice (n = 7) have a significant reduction of brain weight (Fisher’s LSD test, ****p<0.0001) compared to that of the wild-types littermates (n = 7), whereas *Trappc9* m-/p+ mice show a milder but significant reduction (n = 6, Fisher’s LSD test, *p<0.05), and *Trappc9* m+/p- mice have a normal brain weight (n = 7, n.s.). Female brains were used in this analysis. (**B)** *Trappc9* is associated with smaller brain size in adult homozygous knockout mice. X-Y plot shows the percentage of changes relative to the controls (100%) against the *p*-values (in log2 scale) testing the difference in 16-week aged *Trappc9-/-* mice comparing to their wild-type littermates at Lateral 0.72mm section (n = 4 brains in each group, 3 females and 1 male). Yellow dots with numbers represent a total of 12 regions (parameters in bold) that were significantly affected (*p*<0.05, 2-tail Student *t*-test; black dots: n.s.).

One of the most striking features of the patients with *TRAPPC9* loss-of-function mutations is microcephaly [25–31]. Consistent with this disease phenotype, homozygous *Trappc9* deficient mice displayed an 11% reduction in adult brain weight (0.42±0.02g, mean ± SD, n = 6) compared to the wild-type littermates (0.47±0.01g, n = 7). This reduction in brain size exceeds the 3-fold standard deviation threshold [44] of mean brain size commonly used as a benchmark of microcephaly (Fig 4A, *p*<0.0005, Fisher’s LSD test, S5 Data).

To further characterize the brain defect in *Trappc9* deficient mice, we analyzed brain morphology in 16-week old homozygous *Trappc9* knock-out mice and wild-type littermates, assessing 40 brain parameters across 22 distinct brain regions (S2 Table, also see Material and Methods). Compared to wild-type littermates, the total brain area in *Trappc9* ^*-/-*^ mice was decreased by 12.7% (*p* = 0.011), with a 4.1% decrease in width (*p* = 0.036) and 9.8% decrease of height (-9.8%, *p* = 0.014) of the whole brain (n = 4 per genotype, Fig 4B). Significant changes were evident for a further eleven parameters (Fig 4B, S2 Table), showing that both grey and white matter were affected, including a reduction in total area of the cortex (-10.0%, *p* = 0.030), thickness of the primary motor cortex (-11.7%, *p* = 0.020), area and height of the cingulate cortex (-25.7%, *p* = 0.018; -12.1%, *p* = 0.050), area of the thalamus (-15.9%, *p* = 0.017), areas of the corpus callosum (-12.7%, *p* = 0.037) and area of the fimbria of the hippocampus (-17.7%, *p* = 0.048) (Fig 4B). Intriguingly the brain region of greatest reduction was the substantia nigra, showing a 49.1% overall decrease in area in *Trappc9*-/- mice (p = 0.017, Fig 4B).

To assess the importance of imprinting on *Trappc9* function, we next measured brain weight in heterozygous maternal (*Trappc9* ^*m-/p+*^) and paternal (*Trappc9* ^*m+/p-*^) knock-out mice. Consistent with its imprinting, mice lacking the *Trappc9* maternal allele (*Trappc9* ^*m-/p+*^) showed a reduction in brain weight (0.45±0.01g, ≤ -2×SD, p<0.05, two-tailed t-test, n = 6), whereas mice lacking the paternal allele (*Trappc9* ^*m+/p-*^) showed no significant difference in brain weight (0.46±0.03g, p>0.05, two-tailed t-test, n = 7, Fig 4A). *Trappc9* deficient mice therefore exhibit microcephaly with parent-of-origin effects.

### *Trappc9* deficient mice show reduced exploratory activity and impaired social memory

**Fig 5:**
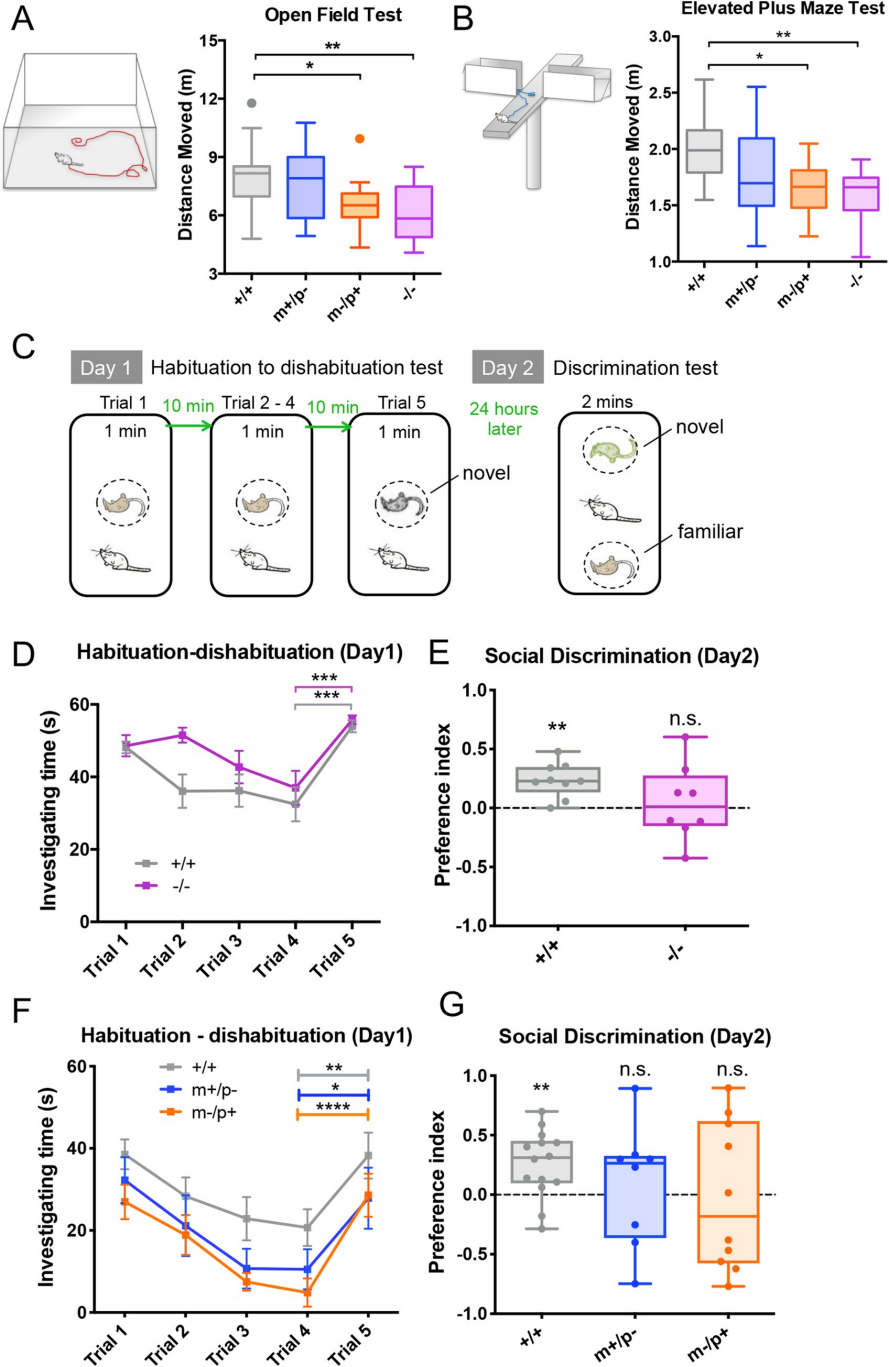
*Trappc9* deficient mice show a parent-of-origin dependent reduction in exploratory activity, and impaired social memory. In (**A)** Open Field Test and (**B)** Elevated Plus Maze Test, mice lacking the maternal (m-/p+, n = 12) or both *Trappc9* alleles (-/-, n = 13) show a significant reduction in exploratory activity compared to mice lacking the paternal allele (m+/p-, n = 12) or their wild-type littermates (+/+, n = 22). One-way ANOVA with Tukey’s *post hoc* test, *p<0.05, **p<0.01. In (**C)** Two-step social recognition test in which mice were assessed for their social learning and memory, **(D)** *Trappc9* homozygous knock-outs (-/-, n = 11) and their wild-type littermates (+/+, n = 10) both showed normal social learning, but (**E)** 24 hours later in the discriminative test, homozygous *Trappc9* ^*m-/p*-^ mice show a significant defect in social recognition as they did not distinguish a familiar mouse from a novel one (one-sample *t*-test, +/+ (n = 9): *p*<0.005, -/- (n = 8): n.s.). Preference Index (1≥ PI ≥- 1) = (sniffing time to the novel stimulus—sniffing time to the familiar stimulus) / (sniffing time to the novel stimulus + sniffing time to the familiar stimulus). (**F)** Maternal (m-/p+, n = 11) and paternal (m+/p+, n = 8) *Trappc9* heterozygous knock-out mice show normal learning abilities compared to wild-types (+/+, n = 15), but (**G)** heterozygous knock-outs show similar but more variable social memory defects (one-sample *t*-test: +/+ (n = 14), *p*<0.01, m+/p- (n = 8), m-/p+ (n = 10): n.s.) Two-way ANOVA with repeat measurement for 1–4 trials: *p*<0.0001 for trial, n.s. for genotype; two-tail *t*-test for 4 and 5 trials, * *p*<0.05, ** *p*<0.01, ***p<0.001. Mice tested in (A) and (B) were males and from (C) to (E) were females.

To assess the functional impact of neuroanatomical defects, we next determined the effect of *Trappc9* deficiency on behavior in the form of exploratory activity and social memory. In a 20-minute Open Field (OF) Test, in which mice were allowed to freely explore an open arena, *Trappc9* ^*-/-*^ mice (n = 13) moved 22% less than their wild-type littermates (n = 22) (*p*<0.01, one-way ANOVA with Tukey’s *post hoc* test, Fig 5A, S10A Fig, S6 Data). Similarly, in a 10-minute Elevated Plus Maze (EPM) Test, where mice freely explored a four-arm elevated platform with open and closed arms, *Trappc9* ^-/-^ mice moved 21% less compared to their wild-type littermates (*p*<0.01, one-way ANOVA with Tukey’s *post hoc* test, Fig 5B, S10B Fig, S6 Data). The reduction in explorative activity was unlikely due to anxiety, as we observed no significant difference in time spent in the periphery and center in OF, or in the closed versus open arms in EPM tests (S11 Fig).

We tested social learning ability using a habituation-dishabituation test [45]. Mice were repeatedly exposed to an anesthetized unfamiliar mouse (trial 1–4), followed by exposure to an unfamiliar mouse (trial 5, Fig 5C, S6 Data). *Trappc9* ^*-/-*^ females habituated in a similar manner to wild-type littermates and showed reduced sniffing time towards the familiar versus the unfamiliar mouse, suggesting normal olfactory detection and short-term social memory (Fig 5D, S6 Data). However, upon re-exposure after 24h, wild-type mice still spent longer sniffing the unfamiliar versus the familiar mouse–suggesting social recognition, whereas *Trappc9* ^*-/-*^ females failed to display a significant preference (*p*>0.05, Fig 5E, S6 Data), indicating impaired social memory. This effect was unlikely to be caused by a defect in recognition or motivation, as *Trappc9* ^*-/-*^ mice showed normal social preference towards conspecifics in a three-chamber sociability test (*p*>0.05, one-way ANOVA with Tukey’s *post hoc* test, S12 Fig). Male *Trappc9* ^*-/-*^ mice showed persistent sniffing towards any anesthetized mouse and could not be assessed using this assay (S13 Fig).

Consistent with a defect in brain development, homozygous *Trappc9* deficient mice showed impaired explorative behavior and social recognition. Intriguingly, in OF and EPM tests, mice lacking the maternal *Trappc9* allele (*Trappc9* ^m-/p+^, n = 12) showed reduced exploratory activity similar to homozygous knock-outs (p<0.05, one-way ANOVA with Tukey’s post hoc test, Fig 5A and 5B), whereas mice lacking the paternal *Trappc9* allele (*Trappc9* ^m+/p-^, n = 12) retained normal levels of exploratory activity. The situation was slightly more complex in the social memory test. With normal olfactory detection and short-term memory (Fig 5F), six out of ten maternal knock-outs (*Trappc9* ^m-/p+^, n = 10) either erroneously preferred the familiar stimulus or showed no difference in long-term social memory test (Fig 5G, orange dots), and paternal knock-outs (*Trappc9* ^m+/p-^, n = 8) showed a similar but milder defect (Fig 5G, blue dots). Thus, *Trappc9* deficient mice showed a parent-of-origin dependent decrease in explorative activity, and an impaired long-term social memory.

### Trappc9 deficient mice develop obesity in a parent-of-origin dependent manner

**Fig 6:**
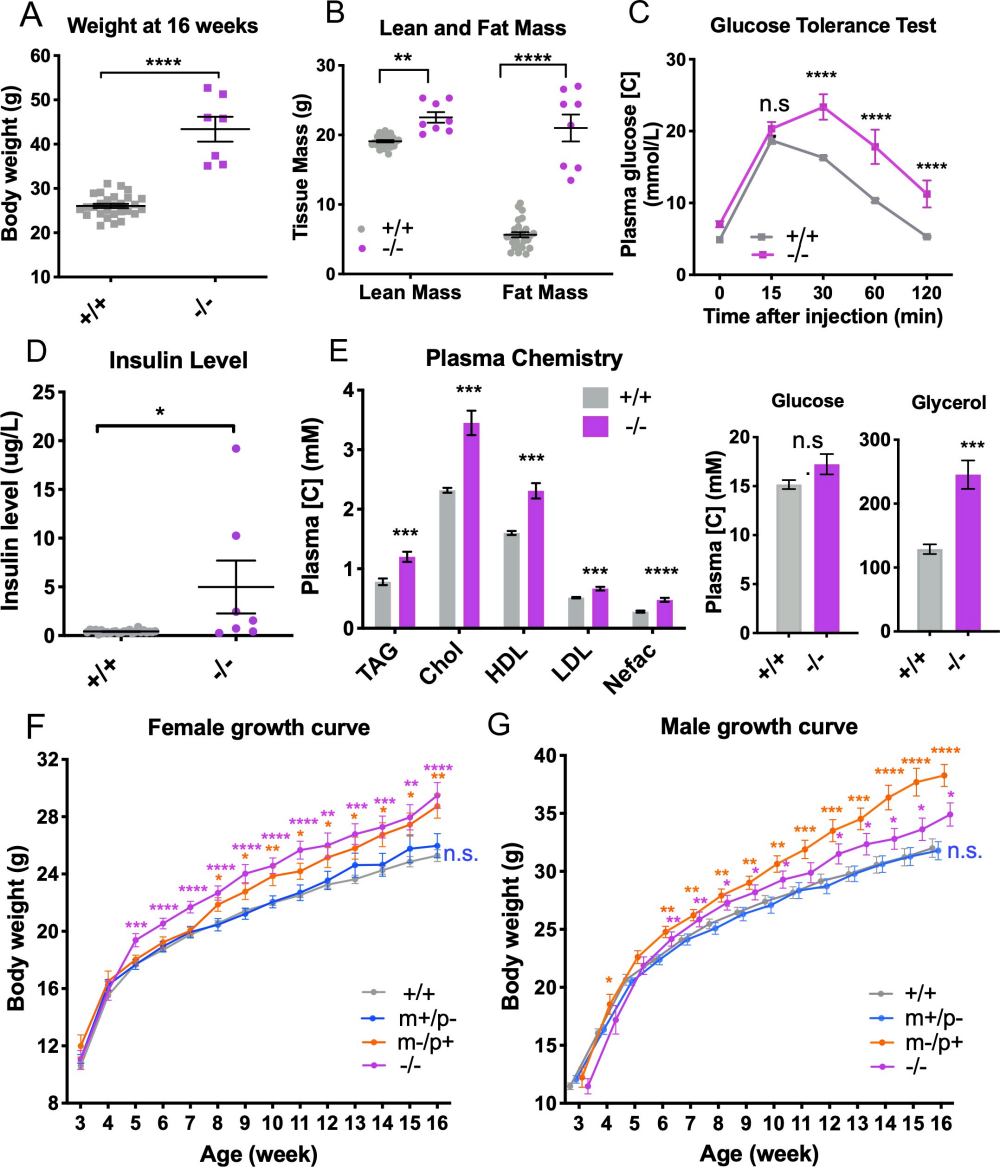
*Trappc9* deficient mice show a parent-of-origin-dependent obesity phenotype. **(A-E)** Female *Trappc9* ^**-/-**^ mice (-/-, n = 8, age = 16 weeks) showed increased body weight (A), increased lean mass and fat mass (B), glucose intolerance (C), increase blood insulin levels (D) and levels of triacylglycerol (TAG), cholesterol (Chol), lipoproteins (HDL & LDL), non-essential fatty acids (Nefac), and glycerol (E), contrasting to wild-type controls (+/+, n = 25–28, 16-week old). Statistical methods for A, B and E: two-tailed unpaired *t*-test; for C: two-way ANOVA with Sidak’s test; for D: two-tailed Mann-Whitney test. (**F-G)** Homozygous (-/-) and heterozygous *Trappc9* knockout mice lacking the maternal-allele (m-/p+) show an increase in body weight in both sexes, whereas heterozygous knockout mice lacking the paternal-allele (m+/p-) have a body weight similar to that of wild-type mice (+/+).Females (**F**, +/+, n = 26–30; m+/p-, n = 9–15; m-/p+, n = 9–13; -/-, n = 9–13), males (**G**, +/+, n = 23–28; m+/p-, n = 10–13; m-/p+, n = 8–9; -/-, n = 11–14). One-way ANOVA with Dunnet’s *post hoc* test was used to test the body weight difference between genotypes at each age, and two-way ANOVA with repeat measurement was used to test the difference between growth curves. *p<0.05, **p<0.01, ***p<0.001, ****p<0.0001.

Besides microcephaly and intellectual disability, obesity is a phenotype commonly observed in TRAPPC9 loss-of-function patients. Indeed, our pipeline screening of homozygous *Trappc9* deficient mice found a significant and steady increase in body weight of both sexes compared to wild-types (~1.5-fold at week 16, n = 7), with a larger effect in female mice (Fig 6A, S14A Fig, S7 Data). In females, this weight gain derives from a marked increase in fat mass and more moderate increase in lean tissue mass (Fig 6B). Furthermore, female mice displayed elevated plasma glucose levels during a glucose tolerance test (Fig 6C), and elevated serum insulin levels (Fig 6D), elevated steady-state plasma levels of triglycerides (TAG), cholesterol (Chol), lipoprotein (HDL/LDL) and glycerol at necropsy (Fig 6E). Male mice also had elevated serum insulin but showed no difference from wild-type in other biochemical markers analyzed (S14B–S14E Fig).

The combination of increased body weight and fat mass in female *Trappc9*-/- mice indicate an obesity phenotype similar to that observed in over half of TRAPPC9 deficient patients [31]. To further assess the effect of *Trappc9* deficiency and its imprinting on body weight, we tracked weight gains of a second, independent, larger cohort of mice containing all four parental genotypes and both sexes. Consistent with our initial finding seen in the pipeline study, homozygous *Trappc9* deficient females (*Trappc9*^*-/-*^) showed a significant increase in weight starting from week 5 after birth (Fig 6F, S8 Data) and cumulatively gained 16% more weight by week 16 (29.49±3.22g) compared to wild-type littermates (25.29±2.16g). Similar weight gains were observed in homozygous *Trappc9* deficient males (*Trappc9* ^*-/-*^, Fig 6G, 11% at week 16, 34.91±3.60g vs. 31.59±3.63g, S8 Data).

Similar to homozygous knock-out mice, we observed a significant increase in the body weight of both male and female heterozygous knock-out mice lacking the maternal *Trappc9* allele (*Trappc9* ^*m-/p+*^) (Fig 6F and 6G). Weight increases in these mice started from week 6 after birth in males and week 8 in females and resulted in a weight gain of respectively 21% (38.3 ± 2.7g) and 13% (28.7 ± 2.5g) at week 16 compared to wild-type littermates (Fig 6F and 6G). In contrast to the maternal *Trappc9* knock-outs, a weight increase was not observed in heterozygous knock-out mice lacking the paternal *Trappc9* allele (*Trappc9* ^*m+/p-*^), whose body weight remained indistinguishable from that of wild-type littermates (male: 31.8 ± 2.8g, n = 13; female: 26.0 ± 3.3g, n = 15). As maternal *Trappc9* knock-out mice (*Trappc9* ^*m-/p+*^*)* retain 30% brain *Trappc9* expression versus 70% in paternal *Trappc9* knock-out mice (*Trappc9* ^*m-/p+*^) mice (Fig 3C), the obesity phenotype positively correlates with the degree of *Trappc9* depletion and therefore with *Trappc9* allelic-biased expression in the brain.

We attempted to determine if loss of *Trappc9* impacts upon food intake, energy expenditure or both by studying mice in an indirect calorimetry system. However, the *Trappc9* homozygous knockout mice lost weight during the acclimatisation periods when singly housed, making it difficult to draw conclusion about the drivers of the obesity phenotype.

### Obesity associated with *TRAPPC9* deficiency may be caused by hyperphagia

Through recruitment to the Deciphering Developmental Disorder (DDD) consortium we had the rare opportunity to examine a 12-year-old child with bi-allelic splice-site mutations in TRAPPC9 (c.2851-1G>C; c.2148+1G>A), regarding her eating behavior and energy intake. This participant had severe global developmental delay with minimal speech, a disordered sleep-wake cycle (sleeping ~3–5 hours a night), stereotyped behaviors and obesity (weight = 57.6kg; height = 146.9 cm; BMI = 27 kg/m^2^; BMI sds = 1.8). Body composition was measured by dual energy x-ray absorptiometry (DEXA); percentage body fat was increased at 45 percent (normal range, 15 to 25 percent). Intriguingly, the child also had a history of severe hyperphagia, showing strong food seeking behavior since infancy (locks required on cupboard doors) and impaired satiety. Her energy intake at an 18MJ *ad libitum* test (meal given after an overnight fast) was significantly greater than that of 10 normal weight and age-matched controls (98 vs 55 +/- 12 KJ/kg lean mass). The basal metabolic rate (BMR) measured by indirect calorimetry was comparable to that predicted based on the basis of age, gender and body composition (4.3 MJ/day). Based on a single case, this suggests an essential role for *TRAPPC9* in normal functioning of the brain feeding circuitry, with deficiency leading to hyperphagia and obesity in human.

## Discussion

In this study, we explored allelic biased gene expression in the mouse olfactory system and identified brain-specific allelic biased expression of the cellular trafficking factor gene *Trappc9*, with 70% of *Trappc9* transcripts in the brain derived from the maternal allele and 30% from the paternal allele. Loss of function mutations in human *TRAPPC9* is a rare hereditary condition that causes a form of intellectual disability, many features of which are recapitulated in the *Trappc9* deficient mouse model described here, including a reduction in brain size (microcephaly), behavioral abnormalities and an increase in fat mass and body weight. We demonstrated the physiological relevance of allelic imbalance in *Trappc9* expression and its brain-specific function, with clear differences observed in adiposity, brain size and behavior dependent on whether deficiency is inherited from the mother or the father. Hence, our study suggests a role for *Trappc9* imprinting in brain development and brain control of energy balance, and provides a new mouse model for *TRAPPC9*-associated intellectual disability.

Genomic imprinting, broadly described as parent-of-origin dependent allelic biased expression, is suggested to be widespread in the human and mouse genome [12]. We here explored brain-specific allelic biased expression by comparing genome-wide, allele-specific expression in the neuronal olfactory bulb (OB) versus the non-neuronal derived main olfactory epithelium (MOE) in mice. The OB shows a robust and reproducible parent-of-origin effect in expression of a number of genes, whereas this bias was observed less frequently in the MOE, consistent with previous studies suggesting enrichment of imprinted gene expression in the brain [13, 19–21].

Besides canonical imprinting with monoallelic expression, we confirmed a new type of imprinted genes with consistent maternal or paternal allelic bias (e.g. *Trappc9*, *H13*), and identified genes of parental allelic bias with high variability across individuals (e.g. *Cyp1a1*, *Gm14097*). Some of these genes are novel (e.g. *Cyp1a1*) whereas others have been characterized with variable results in previous imprinting studies, e.g. *Cdh15*, *Eif2c2* (*Ago2*) and *Trappc9* [4, 5, 35]. P450-type monooxygenase gene *Cyp1a1* (Fig 2A and 2B), does not reside near a known imprinted region and is possibly regulated by a different, stochastic process for its allelic biased expression [46], whereas *Trappc9* shows consistent, maternally-biased, brain-specific allelic biased expression [12, 13] and resides in the characterized *Peg13*-*Kcnk9* imprinting cluster regulated by the intronic long non-coding RNA *Peg13*. Without single-cell level single-molecule imaging data or single-cell allele-specific RNA-sequencing data, we cannot know whether all cells in the relevant regions are allelically biased for *Trappc9* or whether some cells are fully imprinted and others fully biallelic.

Microcephaly and intellectual disability are amongst the most common features of patients with *TRAPPC9* mutations and similar decreases in brain size and behavioral changes are observed in homozygous *Trappc9* deficient mice. Size reductions are observed in multiple regions including the cortex (10–25% reduction), thalamus (-16%), hippocampus (-18%) and most noticeably the substantia nigra (-49%). It is currently difficult to ascribe specific Trappc9-deficiency phenotypes to anomalies in discrete brain regions. Besides a function in cellular membrane trafficking which might be important for neurosecretory cells, particularly those in the hypothalamus, TRAPPC9 is suggested to play a role in NF-κB signaling [23], a pathway critical to neuronal cell differentiation and myelin formation [47]. An impairment in NF-κB–dependent neurite outgrowth might thus underlie defects in brain development and reductions in brain size in general and volume of the cerebral regions and corpus callosum in *Trappc9/TRAPPC9*-deficient mice and human.

One of our most striking observations is that reductions in brain size and exploratory activity in *Trappc9* heterozygous knock-out mice correlate with its allelic biased expression in the brain. *Trappc9*-deficiency phenotypes are thus inherited in the mice lacking the maternal- (*Trappc9* ^*m-/p+*^, 30% remaining Trappc9 expression), but not the paternal *Trappc9* allele (*Trappc9* ^*m+/p-*^, 70% remaining expression). It is currently unclear whether *Trappc9* is imprinted throughout the entire brain or only in specific regions and/or cell types. Future studies will aim to understand the importance of *Trappc9* function and imprinting within individual brain regions and cell types.

Besides microcephaly, obesity is a common feature in TRAPPC9 deficient patients (12/23 patients) [31] and this phenotype is recapitulated in the *Trappc9* deficient mouse model. Like the microcephaly phenotype, the obesity phenotype manifests in a parent-of-origin dependent manner. Heterozygous knock-out mice lacking the maternal allele (*Trappc9* ^*m-/p+*^) show a phenotype similar to the homozygous null mice, whereas those lacking the paternal allele (*Trappc9* ^*m+/p-*^) are phenotypically normal. A dysfunctional imprinted gene network has been associated with an epigenetically regulated bi-model distribution of obesity, triggered by *Trim28* haploinsufficiency, in both mice and human [48]. *Trappc9* was among a group of down-regulated genes that specified the obese state, suggesting a link between *Trappc9* expression and *Trim28*-related epigenetic regulatory mechanisms. As *Trappc9* imprinting occurs specifically in the brain and not in other tissues [12, 13], we reason that this parent-of-origin dependent obesity phenotype is most likely to be driven by disruption of the regulatory pathways in the brain. A potential dysregulation in the neuronal feeding circuitry is reinforced by the clear hyperphagia seen in the TRAPPC9 deficient child with cognitive defects and severe obesity, as it is well recognized that the major regulatory centers for appetitive behavior reside in central nervous system such as the hypothalamus. Future studies including loss of *Trappc9* within selected neuronal circuits and cell types are required to examine the role of *Trappc9* and its imprinting upon the feeding circuits in the brain. With a putative cellular role of *Trappc9* in anterograde vesicular protein trafficking [22], pathways involving the processing and secretion of anorexigenic neuropeptides are likely to be of particular interest.

Sex- specific differences in body weight are common in phenotypic metabolic studies and have been reported in many other model organisms with disruption of the central nervous system based signaling systems; for example, studies based around *TrkB* [49], GABA [50] and *Tmem18* [51]. The mechanisms behind the sex-specific differences in body weight in our study remain to be fully determined, although gonadal derived hormones are likely to be an important factor [52].

Although the *Peg13*-*Kcnk9* imprinting cluster is conserved in humans, imprinting of human *TRAPPC9* remains controversial [32]. All *TRAPPC9*-deficiency patients reported to date carry composite homozygous *TRAPPC9* mutations [27] or compound heterozygous mutations [31]. Even so, if conclusions from our mouse model can be extended, heterozygous carriers of the maternal mutant allele may show subclinical TRAPPC9 related phenotypes, including reduction in brain size, cognitive behavior and obesity, whereas carriers inheriting the paternal mutant allele may not. These effects are easily diluted out in standard genetic analyses in which carrier mutations of paternal/maternal origin are commonly pooled. Separated phenotypic analysis of maternal and parental heterozygotes is recommended in future studies.

In summary, in this study we identified *Trappc9* as a brain-specific imprinted gene, preferentially expressed from the maternally-inherited chromosome. Similar to patients with TRAPPC9-deficiency associated intellectual disability, homozygous *Trappc9* deficient mice show a reduction in brain size (microcephaly), increase in body weight and fat mass (obesity) and behavioral abnormalities. Remarkably, these features are inherited in heterozygous knock-out mice in a parent-of-origin dependent manner that correlates with *Trappc9* allelic biased expression in the brain. Our study yields valuable insight into the molecular pathology underlying human disease and suggests a physiological role for *Trappc9* and its imprinting in brain development, behavior and control of energy homeostasis.

## Material and methods

### Ethics statement

All animal research procedures were approved by the Wellcome Sanger Institute Animal Welfare and Ethics Review Board, by the University of Cambridge Animal Welfare and Ethical Review Body (AWERB), in accordance with UK Home Office regulations, the UK Animals (Scientific Procedures) Act of 1986.

Clinical research studies were conducted as part of a research study protocol that was approved by the Cambridge South Local Research Ethics Committee (reference number 03/103). Clinical investigations were conducted in accordance with the principles expressed in the Declaration of Helsinki. The parents of the child provided written informed consent to her participation, in view of her learning difficulties.

### Mice

Reciprocal crosses of CAST/EiJ and C57BL/6J mice were generated by Duncan Odom Group at the Cancer Research UK Cambridge Institute and studied in the Research Support Facility at Wellcome Sanger Institute. Trappc9<tm1a(EUCOMM)Wtsi> mutant mice were generated by the European Conditional Mouse Mutagenesis Program (EUCOMM) and carried the ‘knockout-first’ allele (tm1a), a *lacZ* reporter-tagged insertion with conditional potential in C57BL6/NTac embryonic stem cells, as described in White et al 2013 [53]. In these knockout mice, *lacZ* expression was below the level of detection in the brain by beta-galactosidase staining and immunohistochemistry. Possible cause of this non-functional reporter may be exon skipping or aberrant splicing over the *lacZ* cassette thus preventing its expression. Parental allelic study cohort of *Trappc9* knock-out mice were generated by heterozygous x heterozygous mating of the *Trappc9* knock-outs in order to track the parent-of-origin of the targeted allele in the offspring. A second cohort of *Trappc9* knock-out mice was used for primary phenotyping pipeline study and consisted of male and female *Trappc9* null mice generated by crossing homozygous with heterozygous knock-outs; they were compared to wild-type control groups of the same sex.

Mice were typically group-housed with 3–5 mice per cage and were given water and diet *ad libitum*, unless otherwise stated. Mice were maintained in a specific pathogen free unit on a 12hr light: 12hr dark cycle (7:30–19:30) and no twilight period, and provided with standard environmental enrichment of a nestlet and a cardboard tunnel. Extended information about the study cohorts and sex of the mice used in experiments can be found in the S1 Text.

### Allelic-specific transcriptomic analysis

To improve power to detect reads that are unique to each strain, Seqnature software [36] was used to construct individualized transcriptomes for the F1 hybrids from the two reciprocal crosses, which increased transcript abundance and read mapping accuracy. CAST SNPs and insertions and deletions (indels) of less than 100 bp were obtained from the Sanger Mouse Genomes Project SNP and indel Release Version 4 and mm10 genome (ftp://ftp-mouse.sanger.ac.uk/REL-1410-SNPs_Indels/). Seqnature was used to construct the CAST-specific genome and gene annotations using Ensembl release 75 (ftp://ftp.ensembl.org/pub/release-75/gtf/mus_musculus) Mus.musculus.GRCm38.75.gtf [54] and to construct CAST transcriptome including all annotated isoforms. B6 and CAST transcriptomes were merged into a single FASTA file and appended labels to track the strain-specific origin of each isoform. A bowtie index was built using bowtie index with B6xCAST diploid transcriptome. RNA-Seq reads were aligned to this diploid transcriptome using bowtie version 1.1.2 with parameters ‘–best’, ‘–strata’, ‘-a’, ‘-m 100’ and ‘-v 3’. These parameters allow us to keep all read alignments with the best alignment score with up to 3 mismatches.

To acquire accurate allelic biased expression, the gene expression was scrutinized through analytic steps summarized in a flowchart (S2 Fig). First, stringent criteria was applied to exclude transcripts with very low expression based on a bimodal distribution of the read frequencies (S3 Fig, at least 25% probability in high expression cohort, based on the methods in [55]). Second, transcripts with low unique reads per gene and those with inconsistent allelic biases across biological replicates were excluded, only transcripts with ≥5 unique reads per gene in both crosses were included. Finally, all unique read counts were adjusted using Expectation-Maximization algorithm for Allele Specific Expression (EMASE, https://github.com/churchill-lab/emase, [37]) to estimate allele-level effective read counts (S4 Fig). To call a gene allelic biased/imprinted, a stringent 0.6 to 0.4 ratio was adopted as a cut-off, which was based on the recommended method in Wang X et.al [39]. In addition, STAR version 2.4 was used to re-align the sequenced reads to mouse reference genome GRCm38 (Ensembl annotation release 78, December 2014), and the parent-of-origin biased expression at single-nucleic polymorphism (SNP) sites were visualized for all the candidate genes using the Integrative Genome Viewer (Broad-MIT, MA, USA).

### Allelic discrimination assay based on RT-qPCR

Custom designed, multiplexed TaqMan MGB probes (Life Technologies, CA) were used to validate the allelic expression bias. They selectively amplified DNA sequences with a single-nucleic polymorphism (SNP) site difference, enabling the detection and quantification of the abundance of cDNAs expressed from two strains within the targeted genes. Targeted SNPs were selected based on the released sequences of CAST/EiJ genome compared to the C57BL/6J genome in the GRCm38 version. Each candidate SNP/Indel location was checked using an in-house MOE RNA-sequencing data and the SNPs were examined in the IGV software (Broad-MIT) using mouse mm10 version of reference genome. Neighboring exons or UTRs were also checked for similar expression level, because primer design may cross exon junctions.

A total of 1 ug purified RNA was reverse transcribed to cDNA using the High Capacity RNA-to-cDNA Kit (Life Technologies, CA). Real-time qPCR reactions (1 uL cDNA in 12 uL total volume) were performed using ABI PRISM 7900HT Fast Real-Time PCR System (Life Technologies, CA) with optimal conditions for thermo-cycling as follows: Step 1, 95C for 10 min; Step 2, 92C for 15 s, 60C for 60 s with Optics; repeated for 40 cycles. Real-time PCR data were analyzed by ABI SDS 2.4.1 software. A standard curve method [56] was used to establish linear relation between log2 ratio of expression level and ΔCt. cDNAs prepared from OB and MOE of inbred CAST/EiJ (n = 4) and inbred C57BL/6J (n = 4), were mixed as the following ratios: 8:1, 4:1, 2:1, 1:1, 1:2, 1:4 and 1:8 (C57 cDNA: CAST cDNA). Standard curves were quantified together with the unknown samples in the same reaction on a 384-well PCR plate and served as internal controls.

### Brain weight assessment and neuroanatomical study

Brain weight of wild-type (+/+), paternal (m+/p-) and maternal (m-/p+) heterozygous and homozygous (-/-) *Trappc9* deficient mice were measured in adult females aged between 11 to 26 weeks. Neuroanatomical study and brain volume analysis were carried out using 4 homozygous (-/-) *Trappc9* deficient mice and 4 matched wild-type littermates (3 females and 1 male *per* group) at 16-week of age. Mouse brain samples were fixed in 4% buffered formalin for 48 hours. Forty brain parameters in 22 distinct brain regions, made of area and length measurements at Lateral 0.72mm, were taken blind to the genotype across one specific sagittal section. Data were analyzed using two-tailed Student *t*-test to determine whether a brain region was associated with neuroanatomical defect or not.

### Primary phenotyping pipeline

The following tests have homozygous mutant mice and wildtype controls, as part of the Sanger MGP phenotyping pipeline. We used the established phenotyping test methods described in the White et al 2013 [53], with the exception that the diet used was Mouse Breeder Diet 5021 and that the pipeline presented here has 4 fewer screens (hair phenotyping, open field, hot plate and stress induced hypothermia tests). Detailed phenotyping procedure can be found in S1 Text.

### Behavioral testing

Individual animals between 8 and 20 weeks of age were tested and monitored by EthoVision XT 8.5 system (Noldus, Netherlands). Mice for behavioral tests had been habituated to the handling of the experimenters for at least two sequential days. All the behavioral experiments were carried out during the light half of the cycle.

Open field test: mice were released at the central point of an open field arena (72 cm × 72 cm × 33 cm) under bright light and were allowed to move freely for 20 minutes. A 36 cm by 36 cm square area in the center of the arena was assigned as “center” area and the rest of the arena was assigned as “border” area. Total distance moved, time spent moving and mean velocity in both center and border areas during the trials were recorded.

Elevated plus maze test: mice were released at the central point of a plus maze (arm size: 30 cm × 5cm × 20cm, height: 50cm above ground) under low light and allowed to freely explore two open arms, two closed arms and the center for 10 minutes. Total distance moved, time spent moving, latency to enter and time spent in three different areas were recorded.

Three-chamber sociability test: A L-shape three-chamber arena (36 cm x 36 cm x 33 cm for each chamber, identical in size) was used in which a “central” chamber connects to “left” and “right” chambers by two small openings at the 12 o’clock and 3 o’clock direction, respectively. The tests were conducted under red light. In the habituation phase, a test mouse was habituated to the central chamber for 5 min and then allowed to freely explore all three empty chambers for 5 min. In the test phase, a metal cylindrical cage (with holes equally distributed on the surface) containing a novel mouse of the same sex and genetic background (C57BL6/NTac) was placed in one of the chambers, whereas an identical but empty cylindrical cage was placed in the other chamber. The test mouse was then released to the central chamber with access to the left and right chambers at will. The duration of stay in each chamber was used to infer the investigating time of each test mouse toward a novel conspecific (mouse in a cage) versus a novel object (empty cage). The chance of a left or a right chamber to contain a mouse was balanced. A preference index (PI) was calculated to score any preference between the two chambers: PI = (duration of stay in mouse chamber- duration of stay in non-mouse chamber)/ (duration of stay in mouse chamber + duration of stay in non-mouse chamber). PI ranges from -1 to 1.

Social recognition test: this paradigm consists of two tests and was carried out in two consecutive days under red light. On day-1, a “habituation-dishabituation” test was used to assess the olfaction detection and memory of a conspecific [45]. After exploring an empty test arena (same size of the home cage) for 10 minutes, the test mouse was presented with an anesthetized mouse on an odorless dish for 1 minute. This stimulus mouse was novel, of a similar weight and age and of same sex and genetic background (C57BL6/NTac from a distant colony). By a 10-mintue interval, the same stimulus mouse was repeatedly presented to the test mouse for four times (trial 1 to 4). In the 5^th^ trial, a different mouse of the same sex but different strain (129S strain) was presented as stimulus. The investigation time, counted as total sniffing time to any part of the body of the stimulus animal was manually scored using a built-in stopwatch in the EthoVision XT. If the test mice showed a habituation to the familiar social stimulus and a dishabituation to the novel social stimulus, then the day-2 test was carried out. On day-2, a discrimination test was carried out to assess the long-term (24-hour) social memory. The test mouse was presented with one familiar mouse (the same mice used on day-1) and one novel mouse (from a third strain, CBA or BALB/c). The sniffing time to each stimulus mouse was recorded as on day-1. A preference index (PI) was used to calculate any preference between novel and familiar stimulus mice, where PI = (sniffing time to the novel stimulus—sniffing time to the familiar stimulus) / (sniffing time to the novel stimulus + sniffing time to the familiar stimulus). PI ranges from -1 to 1.

### Clinical measurements

Weight and height were measured barefoot in light clothing. Whole body dual X-ray absorptiometry (DEXA) (DPX software; Lunar Corp) was used to determine body composition. Ad libitum energy intake was assessed using a 18MJ breakfast meal of known macronutrient content (50% carbohydrate, 30% fat, 20% protein) after an overnight fast; intake was expressed per kilogram of lean body mass measured by DEXA. Basal metabolic rate was determined by indirect calorimetry after a 10 hour overnight fast using an open circuit, ventilated, canopy measurement system (Europa Gas Exchange Monitor; NutrEn Technology Ltd.). After adjustment for body composition, basal metabolic rate was compared to predicted metabolic rate based on age and sex specific equations.

## Supporting information

S1 Text Supplementary methods. (DOCX)

S1 Fig Reciprocal crosses for quantifying the allelic imbalance. Crosses between two distant strains of inbred mice—CAST/EiJ and C57BL/6J - were used to generate F1 generation hybrids with sufficient SNPs to enable accurate quantification of allelic-specific gene expression. CAST: CAST/EiJ (brown mice), B6: C57BL/6J (black mice).
(TIF)

S2 Fig Flowchart view of the allelic-specific expression analysis procedure. MOE RNA sequencing data were shown as an example.
(TIF)

S3 Fig Bimodal distribution of high (green line) and low (red line) mean read counts in each cross and each tissue type. Only transcripts having at least 25% probability to fall into the high read count distribution were used to quantify the allelic-specific expression of each F1 reciprocal across in the olfactory bulb and main olfactory epithelium.
(TIF)

S4 Fig Comparison of allelic expression ratios before (x-axis) and after (y-axis) using EMASE adjustment for multiple mapping. Among a total 11418 genes that was quantified with unique reads, 1369 genes (12%) would have been falsely quantified as allelic imbalance and 212 genes (1.9%) would have been falsely omitted from further analysis without using EMASE (shown only the olfactory bulb data in B6xCAST F1 hybrid).
(TIF)

S5 Fig A breakdown of previous reported imprinting genes in our allelic-specific RNA sequencing analysis. (A) Percentages of expressed, unexpressed and unannotated imprinting genes (a total 151 genes based on ref. 41) in the olfactory bulb (OB) and main olfactory epithelium (MOE). Only the high expressed genes with sufficient SNPs were quantifiable allelic-specific expression. Among the quantified allelic-specific expression, 53 genes in OB and 45 genes in MOE were previous reported to be imprinted or as candidates for imprinting genes (light blue). Known imprinted genes or candidate are based on ref. 41. (B) Further breakdown of known and novel imprinted/ parental biased genes in the quantified allelic-specific expression in two tissues. Brain-derived OB had more known imprinted genes; allelic expression in OB were also paternally dominant.
(TIF)

S6 Fig Gene expression levels of *Trappc9* and *Peg13* in four embryonic stages and eleven adult tissues (wild-type). (A) Trappc9 and (B) *Peg13*, measured by quantitative RT-PCR using a mouse C57 (B6) embryo and tissue cDNA panels (AMSBIO). Expression levels were normalized to β-actin expression.
(TIF)

S7 Fig RNA sequencing of key brain regions in *Trappc9* deficient mice. (A) *Trappc9* transcripts abundance showed 18%, 53% and 74% reduction compared to the wild-type expression level in hippocampus, (B) and decreased by 23%, 63% and 79% of the wild-type expression level in hypothalamus (average expression across 5 females per genotype). (C) Volcano plot shows the 13 differentially expressed genes (highlighted in red, FDR<0.05) in the hippocampus between *Trappc9* -/- and wild-types (+/+). (D) Volcano plot shows the 20 differentially expressed genes (highlighted in red, FDR<0.05) in the hypothalamus between *Trappc9* -/- and wild-types (+/+).
(TIF)

S8 Fig Western blots of Trappc9 protein expression. Trappc9 protein expression was accessed in the wildtype (Wt), paternal knockout (Hetp), maternal knockout (Hetm) and homozygous knockout (Ho). Male brains were used, n = 3 per genotype (A) hypothalamus, (B) olfactory bulb, (C) main olfactory epithelium of *Trappc9* mutant mice and wild-type controls. α-tubulin (A) or GAPDH (B-C) were used as loading controls.
(TIF)

S9 Fig Gene expression levels of the nearby imprinted genes remained stable in the olfactory bulb of *Trappc9* deficient mice. (A) *Knck9* and (B) *Peg13* expression was unchanged by the presence of the tm1a alleles (males, n = 5, p>0.05, one-way ANOVA with Dunnet’s post hoc test against the wild- type group). **(**C) An upstream gene *Chrac1* showed a ~20% upregulation in the OB of maternal heterozygous knockouts but not in the OB of *Trappc9* null mice. (D) Another upstream gene *Ago2* (*Eif2c2*) showed a ~30% upregulation in the OB of *Trappc9* null mice but remained unchanged in the maternal or paternal heterozygous knockouts (* p<0.05, ** p<0.01).
(TIF)

S10 Fig Both male and female *Trappc9* -/- mice showed a reduction of exploration in Open Field Test (OF) and Elevated Plus Maze (EPM) Test. (A) Total distance moved (cm) and time spent moving (s) in the open field. (B) Total distance moved (cm) and time spent moving (s) in the elevated plus maze. Data were analysis by two-way ANOVA (genotype by sex), followed by Sidek’s multiple comparison test between genotypes (+/+ vs. -/-). Male +/+: n = 22, -/-: n = 13; Female +/+: n = 9, -/-: n = 11. “Genotype” factor is highlighted in blue.
(TIF)

S11 Fig Behavioral analysis of Trappc9 deficient mice showed no sign of anxiety in either Open Field Test or Elevated Plus Maze Test. (A) Ratio of time spent in the center zone (total time = 1200s). (B) Ratio of distance moved in the center zone (compared to the total distance moved). (C) Ratio of time spent in the open arms (total time = 600s). (D) Ratio of time spent in the closed arms (total time = 600s). One-way ANOVA with Tukey’s post hoc test. Male mice, n = 22,12,12,13 (+/+, m+/p-, m-/p+ and -/-).
(TIF)

S12 Fig *Trappc9* deficient mice showed normal sociability. (A) In a “L-shape” three-chamber apparatus, mice were allowed to choose between two chambers: one with a caged live mouse versus another with an empty cage. (B) Time spent in the chamber containing another mouse (“mouse chamber”), chamber with an empty cage (“non-mouse chamber”) and the empty chamber that the test mouse was original released into (“central chamber”) were recorded. *Trappc9* homozygous knockout mice (-/-), maternal (m-/p+)/ paternal (m+/p-) heterozygous knockouts and wild-type littermates were compared (males, n = 15,12,11,25 respectively). A preference index (PI) was calculated to score any preference between the two chambers: PI = (duration of stay in the mouse chamber—duration of stay in the non-mouse chamber)/(duration of stay in the mouse chamber + duration of stay in the non-mouse chamber). PI ranges from -1 to 1. One-way ANOVA test with Tukey’s post hoc test: n.s. p>0.05.
(TIF)

S13 Fig Social learning in male wild-type and *Trappc9* -/- mice. Wild-type male mice showed a significant social learning (grey line, **p<0.01, n (+/+) = 10) at Day 1 of the social recognition test, whereas *Trappc9* -/- males (purple line) failed to show such social learning (p>0.05 between Trial 4 and Trial 5, two-tail t-test for 4 and 5 trials, n(-/-) = 9). *Trappc9* -/- males also spent significantly longer time to investigate the stimulus mice compared to the wild-types (two-way ANOVA with repeat measurement: p<0.001 for trial and p<0.05 for genotype), may be caused by minimal change of bedding in their home cage, which was applied to reduce male-male aggression. *Trappc9* -/- males could not be used for Day 2 test due to lack of social learning at Day 1.
(TIF)

S14 Fig Physiological pipeline analysis on *Trappc9* -/- male mice. *Trappc9* null male mice (*Trappc9* -/-, n = 7) showed (A) an increased body weight (*p*<0.05, two-tailed unpaired *t*-test) and (B) an elevated blood insulin level (*p*<0.05, two-tailed Mann-Whitney test) in the phenotyping pipeline analysis, compared to the wild-type controls. This cohort of seven male *Trappc9* ^-/-^ mice showed normal (C) lean and fat mass, (D) glucose tolerance and (E) levels of triacylglycerol (TAG), cholesterol (Chol), lipoproteins (HDL and LDL), non-essential fatty acid (Nefac) and glycerol in the blood (*p*>0.05, two-tailed *t*-test), except for a mild decrease in the blood glucose level (*p<*0.05). Male wild-type controls: n (+/+) = 32–34, *Trappc9* null mice: n(-/-) = 7. n.s. not significant.
(TIF)

S1 Table Strain bias-olfactory receptor genes. A total of 184 olfactory receptor genes (ORGs) in MOE that showed differential expression by strain in the analysis of allelic-specific expression using B6 and CAST reciprocal hybrids.
(XLSX)

S2 Table Neuroanatomical analysis. A list of the 22 distinct brain regions (40 parameters) measured in the study. Yellow highlights the parameters significantly changed in null mice comparing to wild-types.
(XLSX)

S1 Data Allelic bias ratios in the olfactory bulb (OB) and major olfactory epithelium (MOE) of F1 individuals. (XLSX)

S2 Data Parental allelic bias ratios of the candidate genes. (XLSX)

S3 Data Allelic Discriminant Assay validates parental allelic biased gene expression. (XLSX)

S4 Data *Trappc9* mRNA abundance in the mutant mice. (XLSX)

S5 Data Brain weight data. (XLSX)

S6 Data Behavioral experiments data. (XLSX)

S7 Data Phenotyping pipeline metabolism data. (XLSX)

S8 Data Parent-of-origin effect on body weight data. (XLSX)
