# Supplementary material for: Trappc9 deficiency causes parent-of-origin dependent microcephaly and obesity
Source: PLoS Genet. 2020 Sep 2;16(9):e1008916. doi: 10.1371/journal.pgen.1008916 (PMC7467316; doi:10.1371/journal.pgen.1008916)
Supplement: S1 Text — (DOCX) [file pgen.1008916.s001.docx]

**Supplementary Methods**

**Extended information on mice**

*Study cohorts*

Parental allelic study cohort of *Trappc9* knock-out mice was a large cohort consisted of male and female mice of four genotypes: *Trappc9* null, maternal and paternal *Trappc9* heterozygous mutant and their wild-type littermates. These mice were generated by heterozygous x heterozygous mating of the *Trappc9* knock-outs in order to track the parent-of-origin of the targeted allele in the offspring.

A second cohort of *Trappc9* knock-out mice was used for primary phenotyping pipeline study and consisted of male and female *Trappc9* null mice generated by crossing homozygous with heterozygous knock-outs; they were compared to wild-type control groups of the same sex.

Both cohorts of mice were given water and diet *ad libitum*, unless otherwise stated. Diet was Breeders chow (Mouse Breeder Diet 5021, 9% crude fat content, 21% kcal as fat, 0.276ppm cholesterol, Labdiet, London, UK). Mice were maintained in a specific pathogen free unit on a 12hr light: 12hr dark cycle (7:30-19:30) and no twilight period. The ambient temperature was 21 ± 2C and the humidity was 55 ± 10%. Mice were typically group-housed with 3-5 mice per cage. In addition to Aspen bedding substrate, standard environmental enrichment of a nestlet and a cardboard tunnel were provided. All the procedures were approved by the Wellcome Sanger Institute Animal Welfare and Ethics Review Board in accordance with UK Home Office regulations, the UK Animals (Scientific Procedures) Act of 1986.

A third cohort of *Trappc9* knock-out mice was used in studies carried out by the Medical Research Council Metabolic Diseases Unit (MRC MDU). They were maintained in a 12-hour light/12-hour dark cycle (lights on 0700–1900), temperature-controlled (22°C) facility, with ad libitum access to food (RM3(E) Expanded chow, Special Diets Services, UK) and water. For all cohorts, both sexes were studied unless stated otherwise. All studies were again performed in accordance with UK Home Office Legislation regulated under the Animals (Scientific Procedures) Act 1986 Amendment, Regulations 2012, following ethical review by the University of Cambridge Animal Welfare and Ethical Review Body (AWERB).

*Sex of the mice used in experiments*

Data of the sex in the parentheses were included in the supplementary figures.

| Experiment | Sex |
| --- | --- |
| Allelic-specific RNA sequencing analysis on F1 hybrids | female |
| Allelic discrimination qRT-PCR assay on F1 hybrids | female |
| qRT-PCR assay and western blotting on *Trappc9* knockouts | male |
| Primary phenotyping pipeline | female (male) |
| Parent-of-origin effect on body weight | male and female |
| Parent-of-origin effect on behavior | male (female) |
| Brain weight analysis  Neuroanatomical study | female  female + male |
| Brain regional RNA sequencing on *Trappc9* knockouts | female |

**RNA sequencing on F1 hybrids**

Five female offspring from F1 generation of CAST/EiJ x C57BL/6J cross and five female offspring from F1 generation of C57BL/6J x CAST/EiJ cross, both at 14 weeks of age, were used to dissect the olfactory bulb and the main olfactory epithelium. Tissues were frozen on dry ice and homogenized in lysis solution (10 uL β-mercaptoethanol in 1 mL buffer RLT) and RNAs were extracted using the RNeasy Mini Kit (Qiagen) following standard protocol for animal tissues. A total of 5 ug RNA were used to produce libraries for multiplexed sequencing using Illumina Truseq protocol. Pair-end sequencing with a read length of 200-240 bp was carried out using Illumina HiSeq 2000.

**Quantitative RT-PCR assay**

TaqMan Gene Expression assays were carried out by ABI PRISM 7900HT Fast Real-Time PCR System (Life Technologies, CA) using standard manufacturer’s protocol. RNA samples were extracted from five males of 14 week of age in three *Trappc9* mutant genotypes and their wild-type littermates. TaqMan probes and primers were predesigned by the manufacturer (assay ID listed in the table below). For tissue-wide gene expression of *Trappc9* and *Peg13* (S6 Fig), mouse C57 embryo and tissue cDNA panels (AMSBIO) were used to access four embryonic stages (fetus) and eleven tissue types (adult). Mean cycle threshold (Ct) values were obtained from three technical replicates and each normalized to *Actb* expression using the Relative Quantity (ΔΔCt) method. Relative quantity (RQ) values were calculated using the formula RQ = 2^−ΔΔCt^.

| Gene Symbol | Gene Name | Assay ID | Mix Conc. | Target Exons |
| --- | --- | --- | --- | --- |
| Trappc9 | trafficking protein particle complex 9 | Mm01201387_m1 | 20x | 6 |
| Kcnk9 | potassium channel, subfamily K, member 9 | Mm02014295_s1 | 20x | 2 |
| Chrac1 | chromatin accessibility complex 1 | Mm00459710_m1 | 20x | 1 |
| Ago2 | argonaute RISC catalytic subunit 2 | Mm03053414_g1 | 20x | 19 |
| Peg13 | paternally expressed 13 | Mm03456085_s1 | 20x | 1 |
| Actb | actin, beta | Mm00661485_m1 | 20x | 3 |

**Genotyping**

**
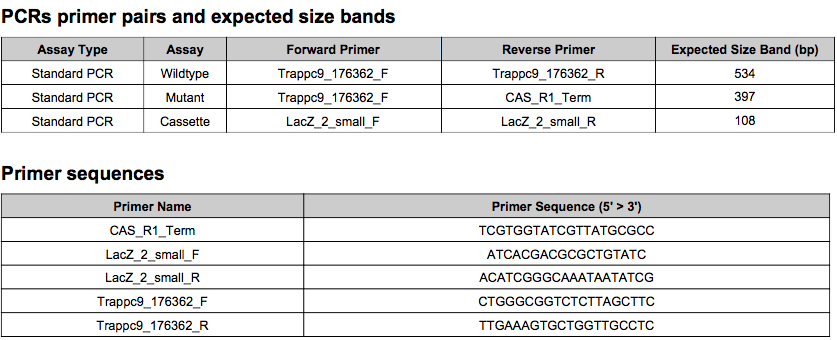
**

**Western blotting**

Male *Trappc9* mutant mice and wild-type littermates (27-30 weeks of age, n=3 per genotype) were sacrificed and the olfactory bulb (OB) and main olfactory epithelium (MOE) were dissected and stored at -80 °C. Protein extracts from OB and MOE were prepared in 200 μl RIPA buffer (pH 8, 50 mM Tris, 150mM NaCl, 1% NP40, 0.5% Nadeoxycholate, 0.1% SDS) supplemented with proteinase inhibitor cocktail (Sigma Aldrich) using a Fast-Prep 24 Homogenizer. The tissue lysates were analyzed on 4%–12% BIS–Tris-acetate gels, incubated and blots were stained with rat monoclonal anti-alpha tubulin (Yl1/2) (1:1000, sc-53029, Santa Cruz Biotechnology) and rabbit anti-Trappc9 (1:800, #16014, ProteinTech), followed by anti-rat (1:8000) and anti-rabbit (1:2000) appropriate secondary antibodies (Santa Cruz Biotechnology) and visualized using Super signal west dura (Thermo Scientific). Trappc9 protein expression was densitometrically analyzed using ImageJ software and alpha-tubulin was used as a loading control.

**RNA sequencing on *Trappc9* mutant mice**

Brain tissues of hippocampus and hypothalamus were collected from female mice of each *Trappc9* genotype (+/+, m+/p-, m-/p+ and -/-, n=5 per group, age range from 11-26 weeks). RNA extraction and library preparation were the same as above RNA sequencing experiments. Read counts of mutant mice are analyzed and tested against +/+ mice by brain regions.

**Primary phenotyping pipeline**

The following tests have homozygous mutant mice and wildtype controls, as part of the Sanger MGP phenotyping pipeline. We used the established phenotyping test methods described in the White et al 2013, with the exception that the diet used was Mouse Breeder Diet 5021 and that the pipeline presented here has 4 fewer screens (hair phenotyping, open field, hot plate and stress induced hypothermia tests).

Weight: We tracked the body weight changes of seven male and seven female mutants (*Trappc9* -/-) from 4 weeks of age. Mice of both genders are weighed at regular intervals between 4 and 16 weeks of age.

Body Composition (DEXA): At week 14, mice were anaesthetized with ketamine hydrochloride (110 mg/kg, Ketaset, Fort Dodge Animal Health)/xylazine hydrochloride (11 mg/kg, Rompun, Bayer Animal Health), imaged on a dual energy X-ray absorptiometry machine (Lunar PIXImus II) and analysed with Lunar PIXImus2 2.1 software (GE Lunar, Madison, WI). Quality control was performed using a calibrated phantom before imaging. This generates an image of the entire mouse and provides bone mineral and body composition data. Mice were examined by both DEXA, X-ray (for skeletal abnormalities) and Auditory Brainstem Response (ABR) while anaesthetized. No phenotype was detected for X-Ray or ABR.

Glucose Tolerance (ip): At week 13, mice were single housed and fasted overnight (maximum of 16 hours) before the glucose tolerance test. Approximately 0.5mm of the tail tip was removed and a fasting blood sample is taken before a bolus of glucose (2g/kg) is administered by intra-peritoneal injection. Blood samples are tested for glucose concentration (Accu-Check Aviva, Roche) at 15, 30, 60 and 120 minutes following the glucose administration, and data presented as plasma glucose concentration.

Comprehensive Plasma Chemistry Panel: Samples for clinical chemistry are routinely taken as part of the necropsy of the mice at week 16. Blood was collected from animals in the random-fed state between 08:30 and 10:30. Mice were anesthetized using 100 mg/kg Ketamine and 10 mg/kg Xylazine and blood was collected into heparinized paediatric tubes (Kabe Labortechnik GmbH, Numbrecht, Germany) using the retro-orbital route, followed by heart removal. Heparinized whole-blood samples were centrifuged at 5,000 rcf for 10 min at 4⁰C, and the plasma was collected and stored at 4⁰C until analysis, always within 1 hour of collection. Plasma variables were assessed at room temperature using an Olympus AU400.

**Parent-of-origin effect on body weight**

Body weight of wild-type (+/+), paternal (m+/p-) and maternal (m-/p+) heterozygous and homozygous (-/-) *Trappc9* deficient mice of both sexes were fed Breeders Chow (Mouse Breeder Diet 5021, 9% crude fat content, 21% kcal as fat, 0.276ppm cholesterol, Labdiet, London, UK), same as the rest of the colonies, and weighed weekly at light cycle between 3 and 16 weeks of age.

**Statistics and software**

The sequencing analysis were using R (version 3.2.2) and were detailed in respective methods. The expression heatmaps were made in R using “pheatmap” package and the distribution analysis on sequencing reads were used “mixtool” package. *Trappc9* expression, adult body and brain weight, physiological data and behavioral data across genotypes were tested by respective statistical methods detailed in the main text, using GraphPad Prism 7 for Mac (La Jolla, CA, USA). Neuroanatomical data were processed in Microsoft Excel for Mac (version 15.25). The entire mouse phenotyping pipeline was analyzed using PhenStat (Kurbatova et al, 2015) and have been described in previously publication (Karp et al, 2012).

**Data availability and software access**

RNA-sequencing studies are accessible at European Nucleotide Archives (https://www.ebi.ac.uk/ena)

PRJEB5160: Detecting parent-of-origin allelic expression bias in mouse olfaction system;

PRJEB14815: The transcriptomic effect of brain specific allelic imbalance of Trappc9.

SEQNATURE software: <http://cgd.jax.org/tools/Seqnature.shtml>

EMASE package for R: <https://github.com/churchill-lab/emase>

Catalogue of Parent of Origin Effect (University of Otago, New Zealand): <http://igc.otago.ac.nz>

Mousebook database (Medical Research Council): <http://www.mousebook.org/mousebook-catalogs/imprinting-resource>

Allen Mouse Brain Atlas: http://mouse.brain-map.org

**Supplementary methods reference:**

White, J.K. et al. (2013). Genome-wide generation and systematic phenotyping of knockout mice reveals new roles for many genes. Cell *154*:452–464.

Sánchez-Andrade, G., Kendrick, K.M. (2011). Roles of α- and β-estrogen receptors in mouse social recognition memory: effects of gender and the estrous cycle. Horm Behav. *59*, 114-22

Kurbatova, N., Mason, J. C., Morgan, H., Meehan, T. F., Karp, N. A. (2015). PhenStat: A Tool Kit for Standardized Analysis of High Throughput Phenotypic Data. PloS ONE *10*, e0131274.

Karp, N. A., Melvin, D., Sanger Mouse Genetics, Mott, R. F. (2012). Robust and sensitive analysis of mouse knockout phenotypes. PloS ONE *7*, e52410.
