# Supplementary figures and images for: Trappc9 deficiency causes parent-of-origin dependent microcephaly and obesity
Source: PLoS Genet. 2020 Sep 2;16(9):e1008916. doi: 10.1371/journal.pgen.1008916 (PMC7467316; doi:10.1371/journal.pgen.1008916)

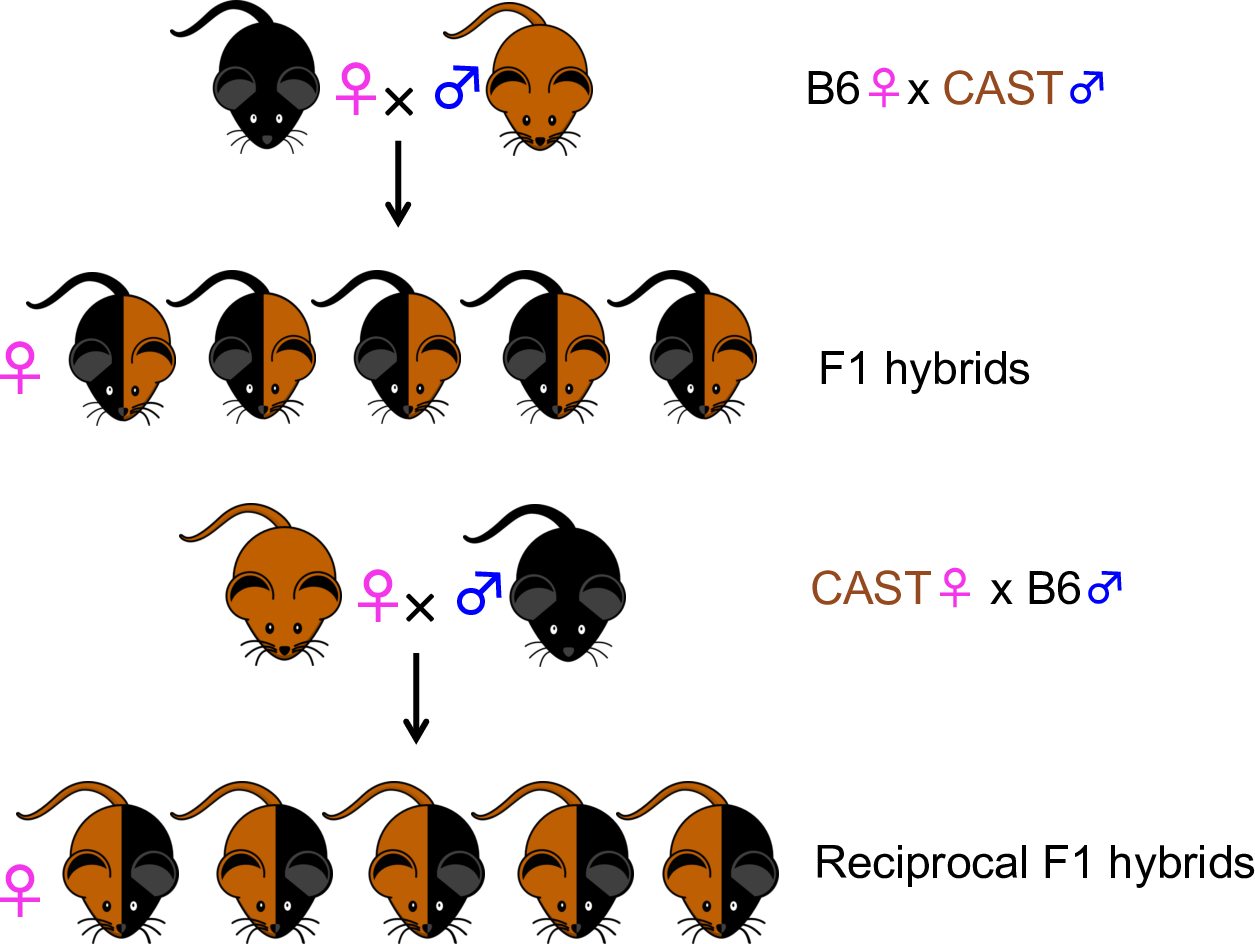

Supplement: S1 Fig — Crosses between two distant strains of inbred mice—CAST/EiJ and C57BL/6J - were used to generate F1 generation hybrids with sufficient SNPs to enable accurate quantification of allelic-specific gene expression. CAST: CAST/EiJ (brown mice), B6: C57BL/6J (black mice). (TIF) [file pgen.1008916.s002.tif]

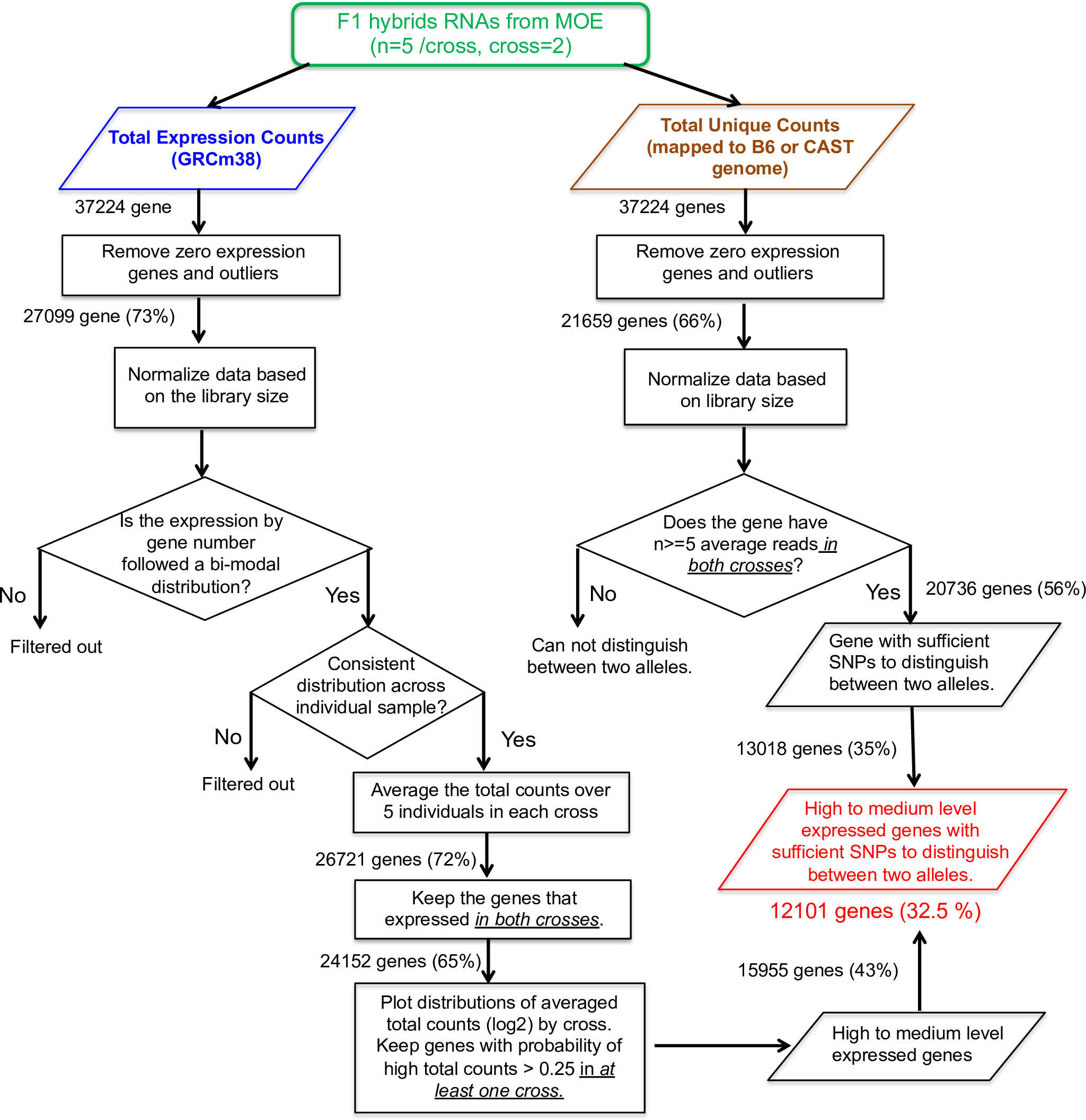

Supplement: S2 Fig — MOE RNA sequencing data were shown as an example. (TIF) [file pgen.1008916.s003.tif]

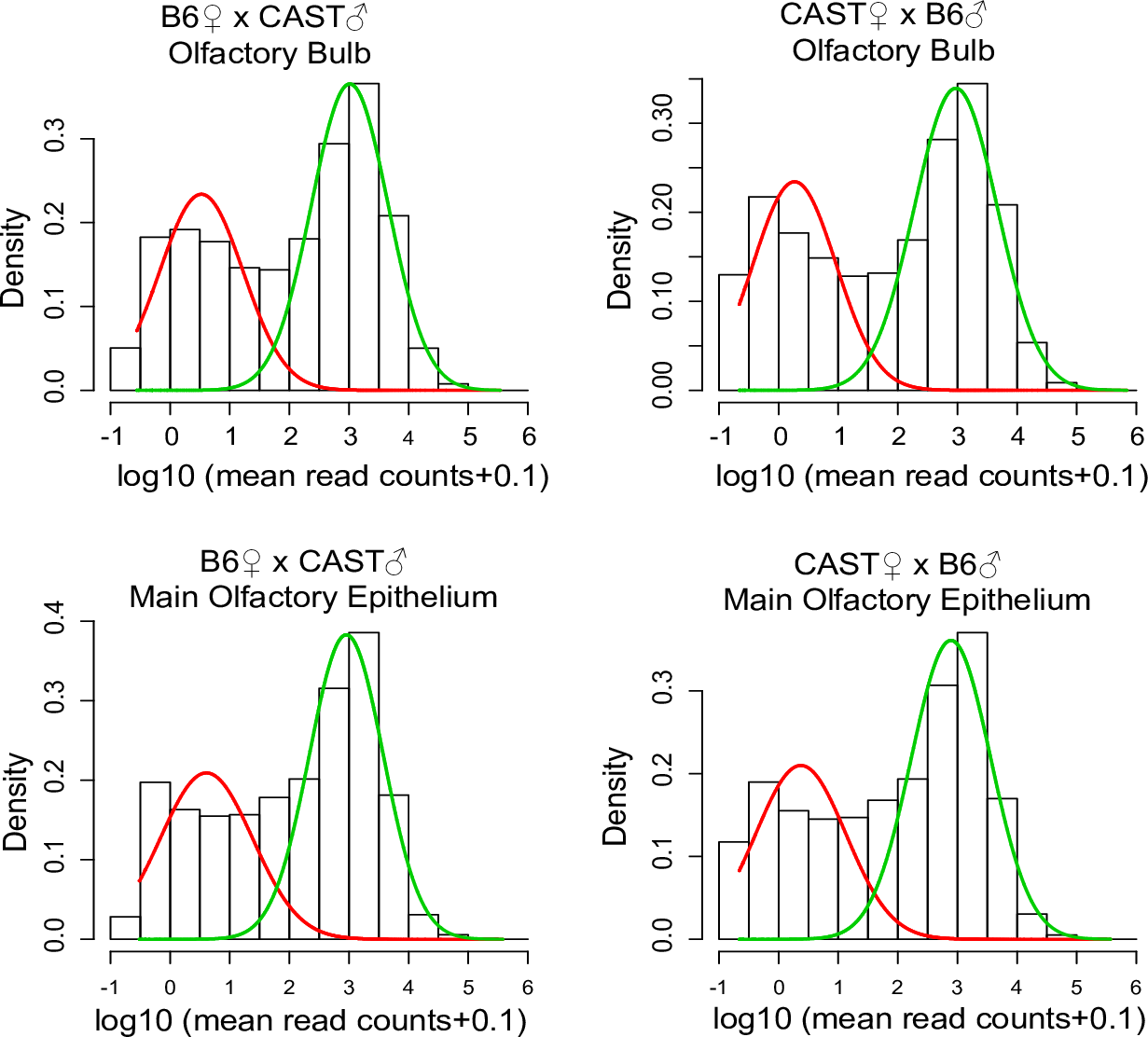

Supplement: S3 Fig — Only transcripts having at least 25% probability to fall into the high read count distribution were used to quantify the allelic-specific expression of each F1 reciprocal across in the olfactory bulb and main olfactory epithelium. (TIF) [file pgen.1008916.s004.tif]

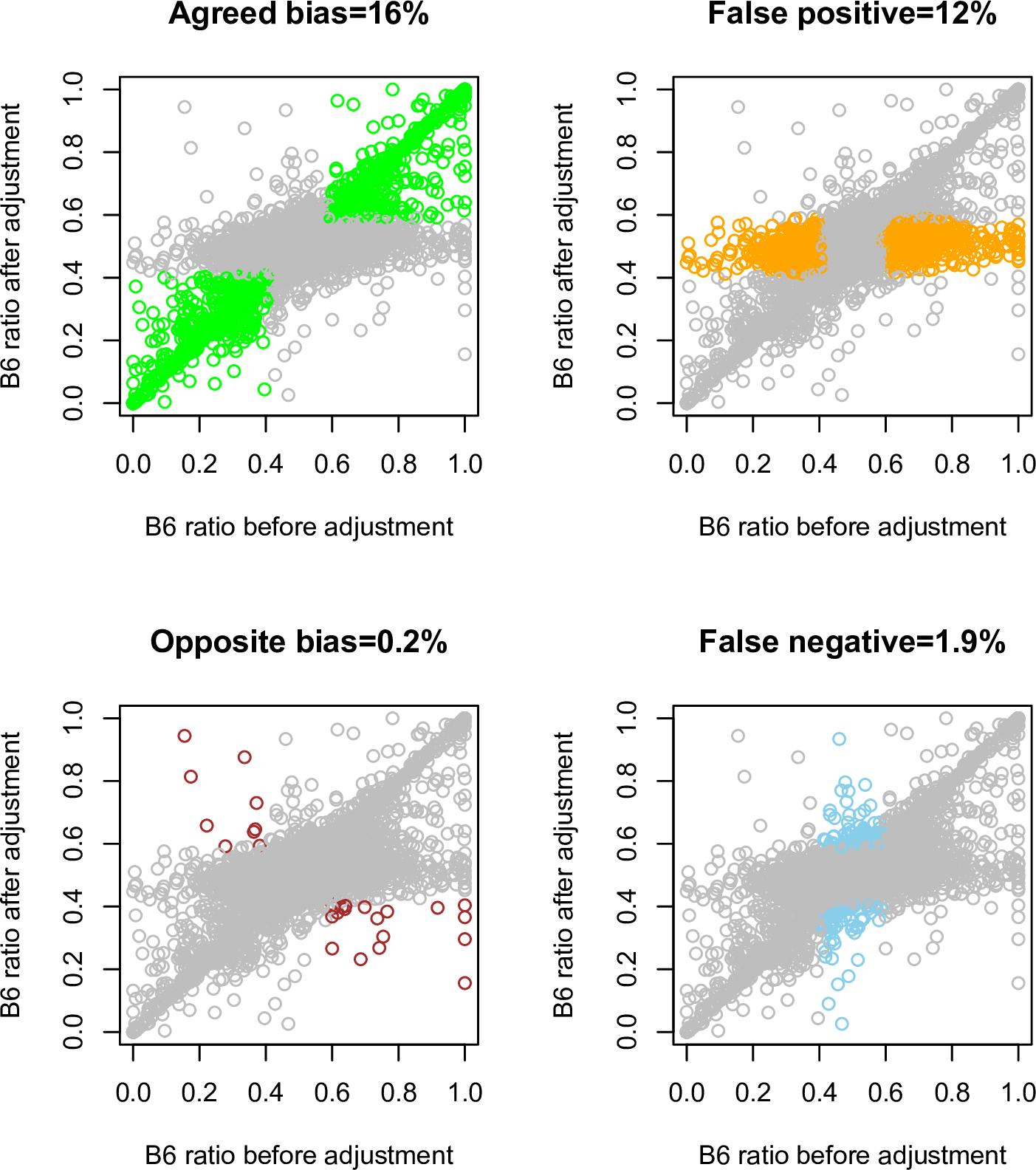

Supplement: S4 Fig — Among a total 11418 genes that was quantified with unique reads, 1369 genes (12%) would have been falsely quantified as allelic imbalance and 212 genes (1.9%) would have been falsely omitted from further analysis without using EMASE (shown only the olfactory bulb data in B6xCAST F1 hybrid). (TIF) [file pgen.1008916.s005.tif]

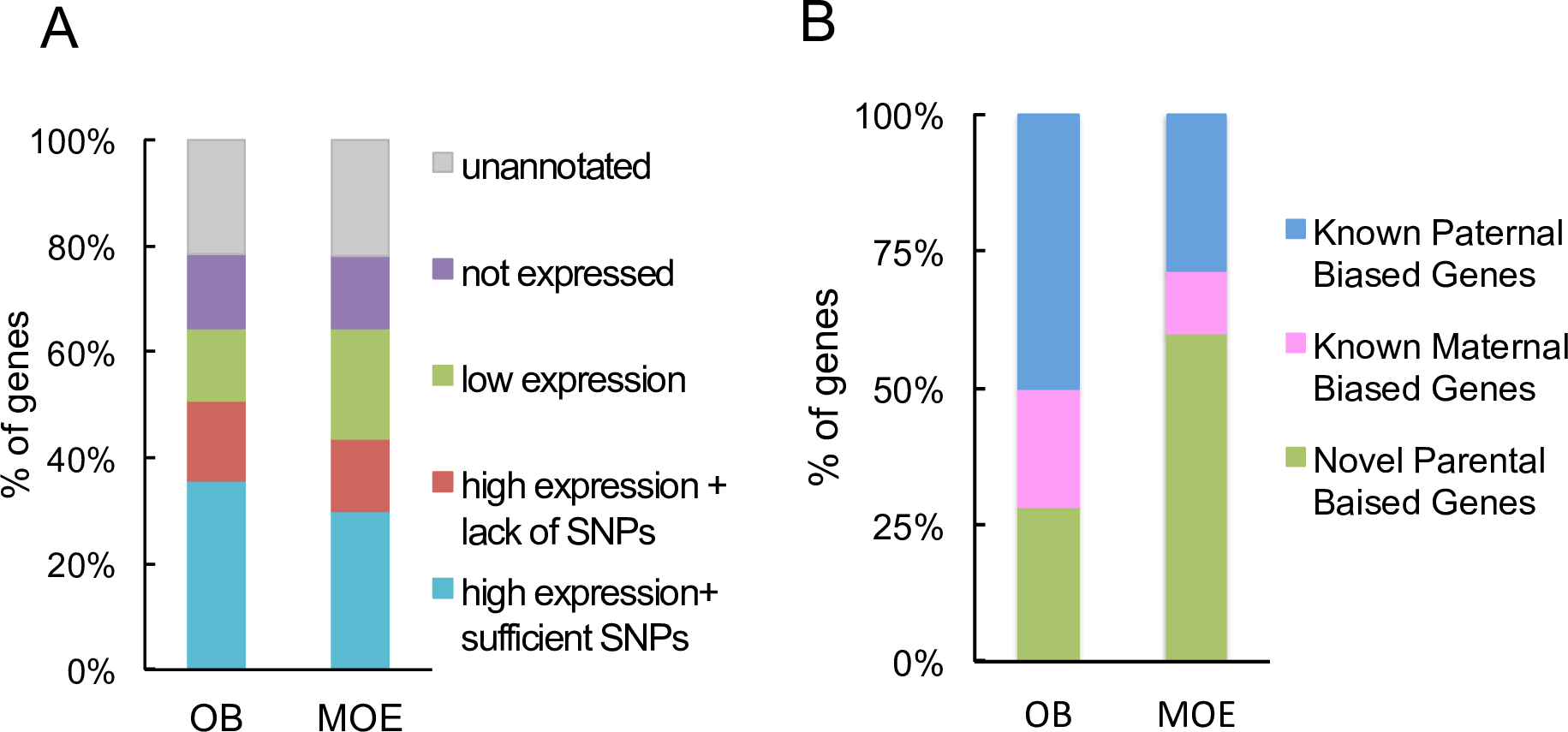

Supplement: S5 Fig — (A) Percentages of expressed, unexpressed and unannotated imprinting genes (a total 151 genes based on ref. 41) in the olfactory bulb (OB) and main olfactory epithelium (MOE). Only the high expressed genes with sufficient SNPs were quantifiable allelic-specific expression. Among the quantified allelic-specific expression, 53 genes in OB and 45 genes in MOE were previous reported to be imprinted or as candidates for imprinting genes (light blue). Known imprinted genes or candidate are based on ref. 41. (B) Further breakdown of known and novel imprinted/ parental biased genes in the quantified allelic-specific expression in two tissues. Brain-derived OB had more known imprinted genes; allelic expression in OB were also paternally dominant. (TIF) [file pgen.1008916.s006.tif]

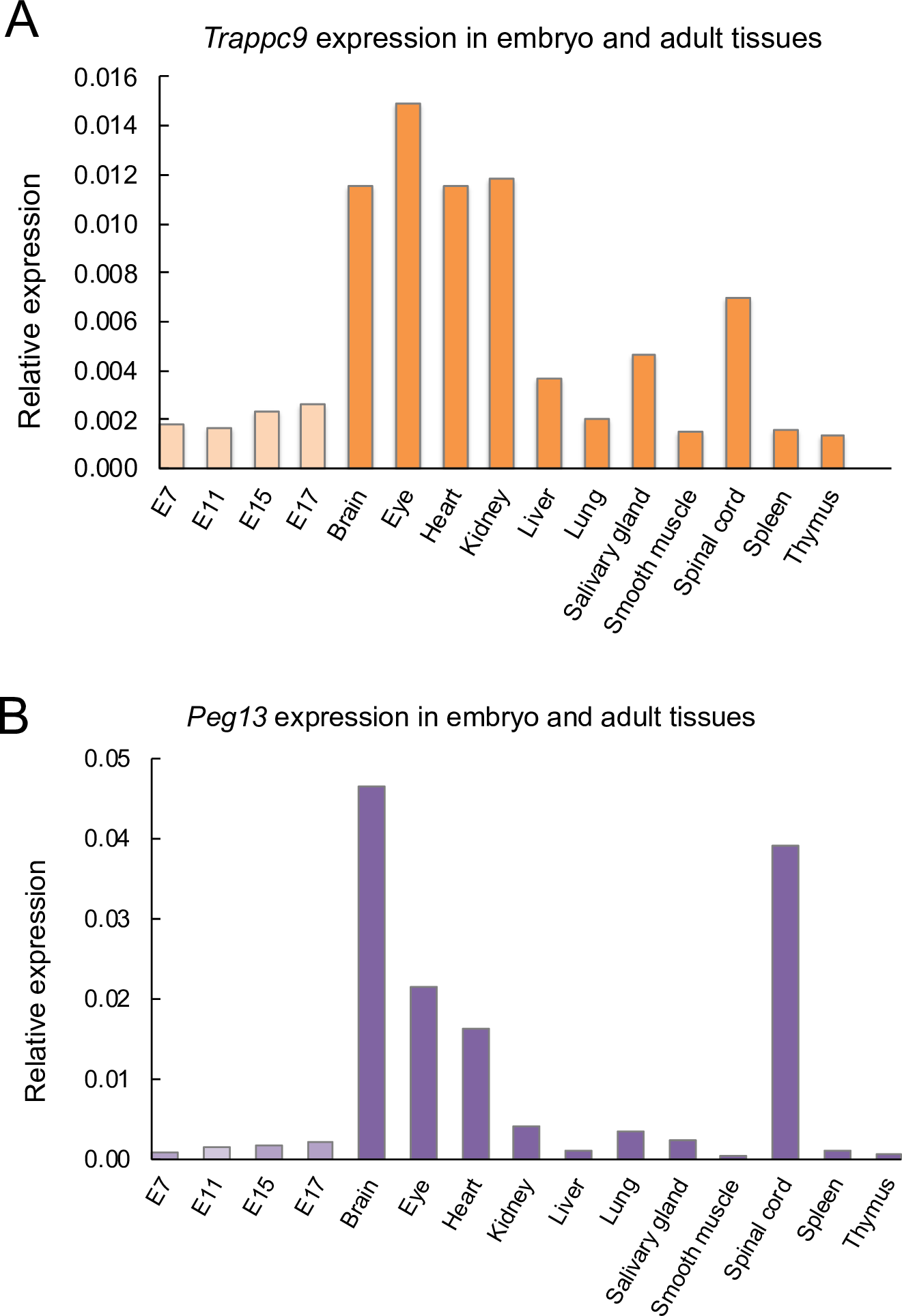

Supplement: S6 Fig — (A) Trappc9 and (B) Peg13, measured by quantitative RT-PCR using a mouse C57 (B6) embryo and tissue cDNA panels (AMSBIO). Expression levels were normalized to β-actin expression. (TIF) [file pgen.1008916.s007.tif]

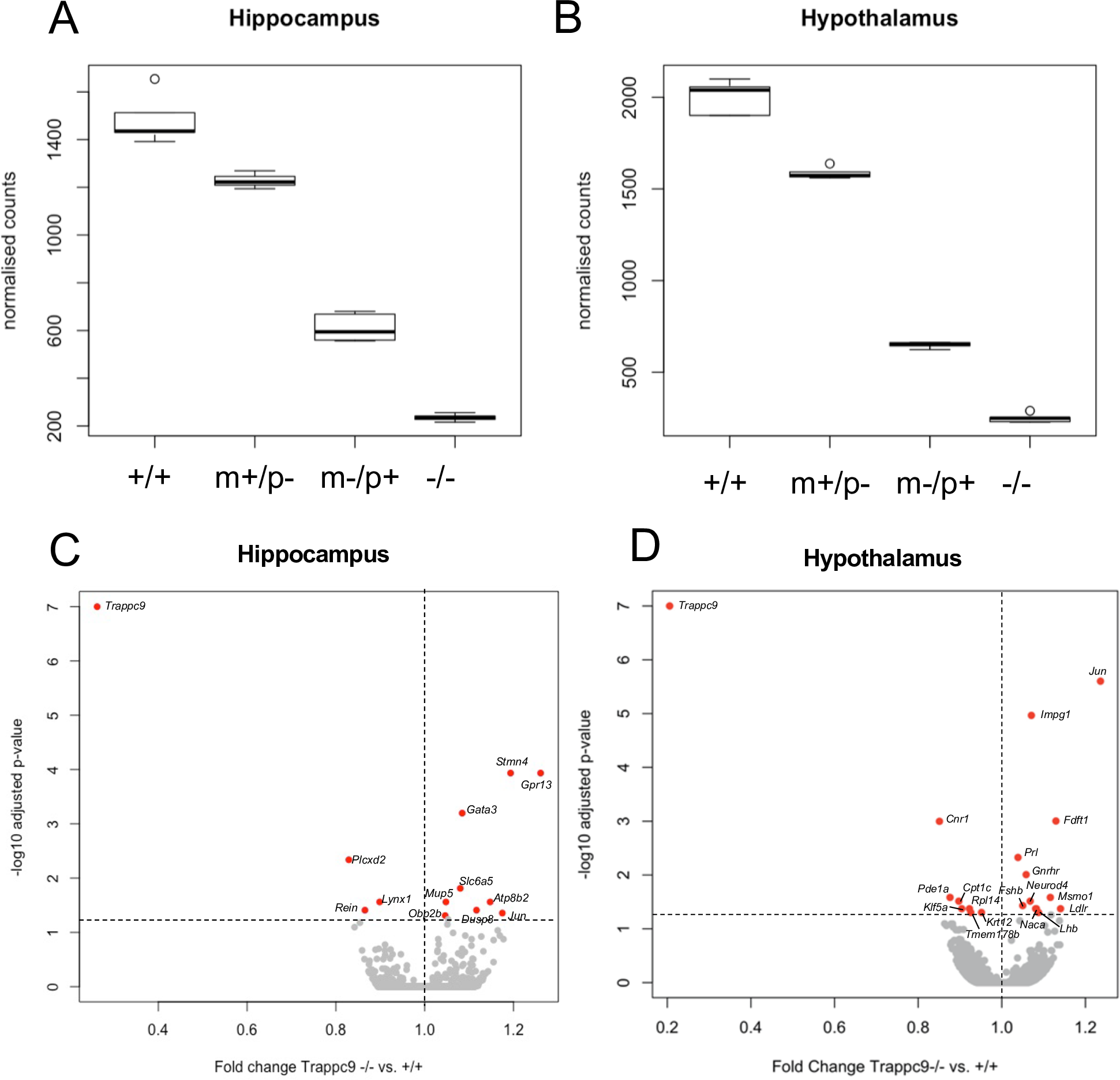

Supplement: S7 Fig — (A) Trappc9 transcripts abundance showed 18%, 53% and 74% reduction compared to the wild-type expression level in hippocampus, (B) and decreased by 23%, 63% and 79% of the wild-type expression level in hypothalamus (average expression across 5 females per genotype). (C) Volcano plot shows the 13 differentially expressed genes (highlighted in red, FDR<0.05) in the hippocampus between Trappc9 -/- and wild-types (+/+). (D) Volcano plot shows the 20 differentially expressed genes (highlighted in red, FDR<0.05) in the hypothalamus between Trappc9 -/- and wild-types (+/+). (TIF) [file pgen.1008916.s008.tif]

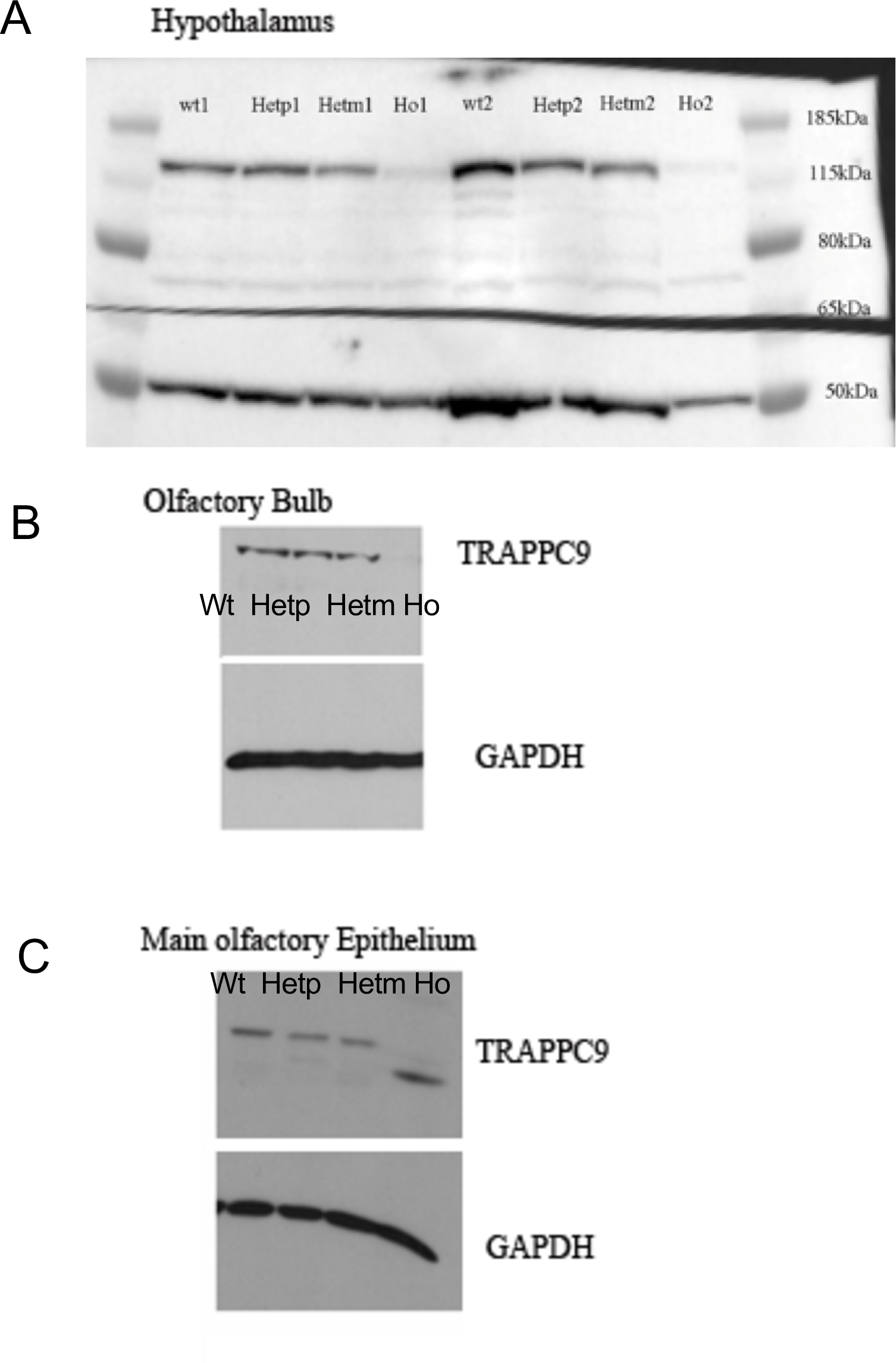

Supplement: S8 Fig — Trappc9 protein expression was accessed in the wildtype (Wt), paternal knockout (Hetp), maternal knockout (Hetm) and homozygous knockout (Ho). Male brains were used, n = 3 per genotype (A) hypothalamus, (B) olfactory bulb, (C) main olfactory epithelium of Trappc9 mutant mice and wild-type controls. α-tubulin (A) or GAPDH (B-C) were used as loading controls. (TIF) [file pgen.1008916.s009.tif]

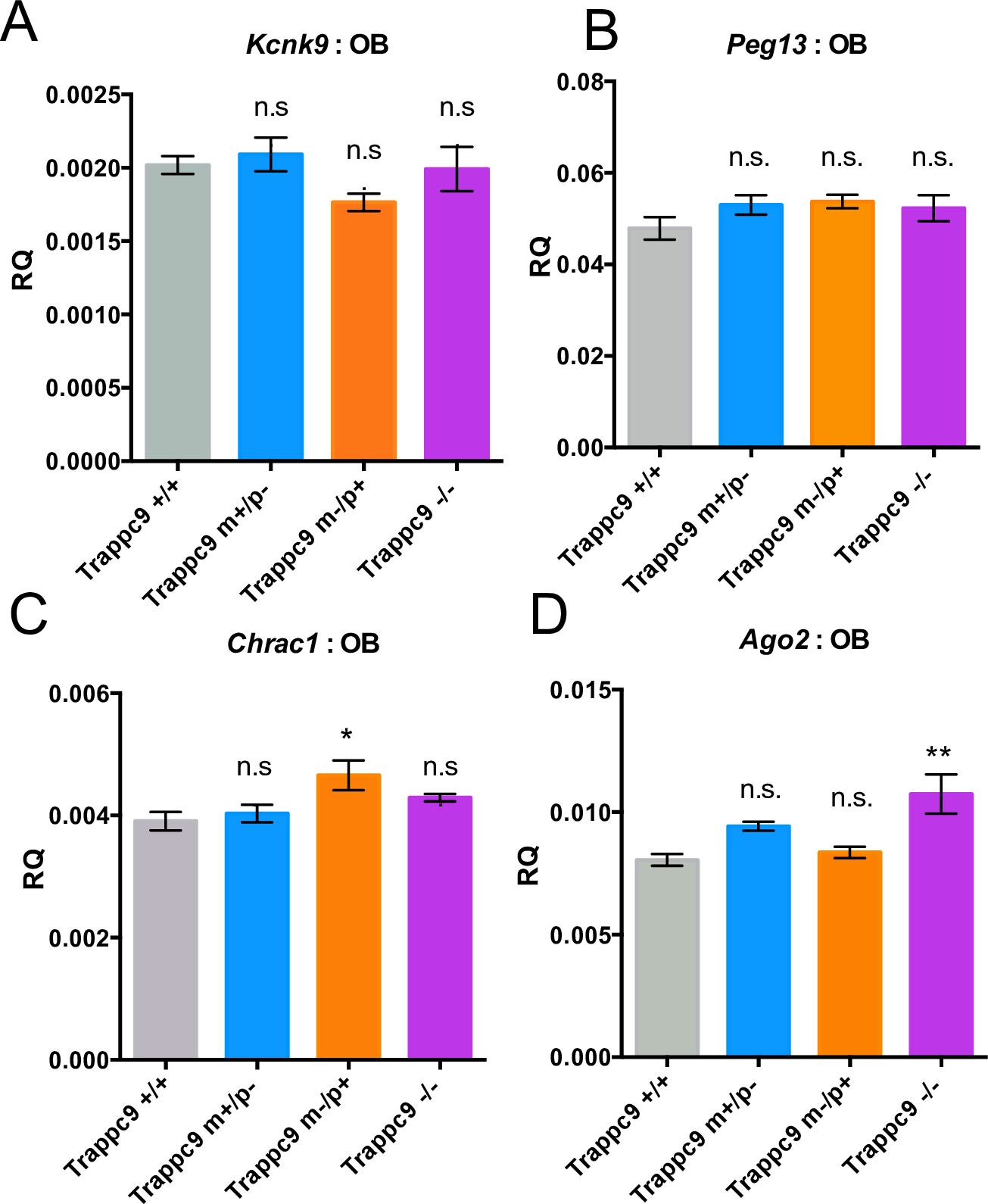

Supplement: S9 Fig — (A) Knck9 and (B) Peg13 expression was unchanged by the presence of the tm1a alleles (males, n = 5, p>0.05, one-way ANOVA with Dunnet’s post hoc test against the wild- type group). (C) An upstream gene Chrac1 showed a ~20% upregulation in the OB of maternal heterozygous knockouts but not in the OB of Trappc9 null mice. (D) Another upstream gene Ago2 (Eif2c2) showed a ~30% upregulation in the OB of Trappc9 null mice but remained unchanged in the maternal or paternal heterozygous knockouts (* p<0.05, ** p<0.01). (TIF) [file pgen.1008916.s010.tif]

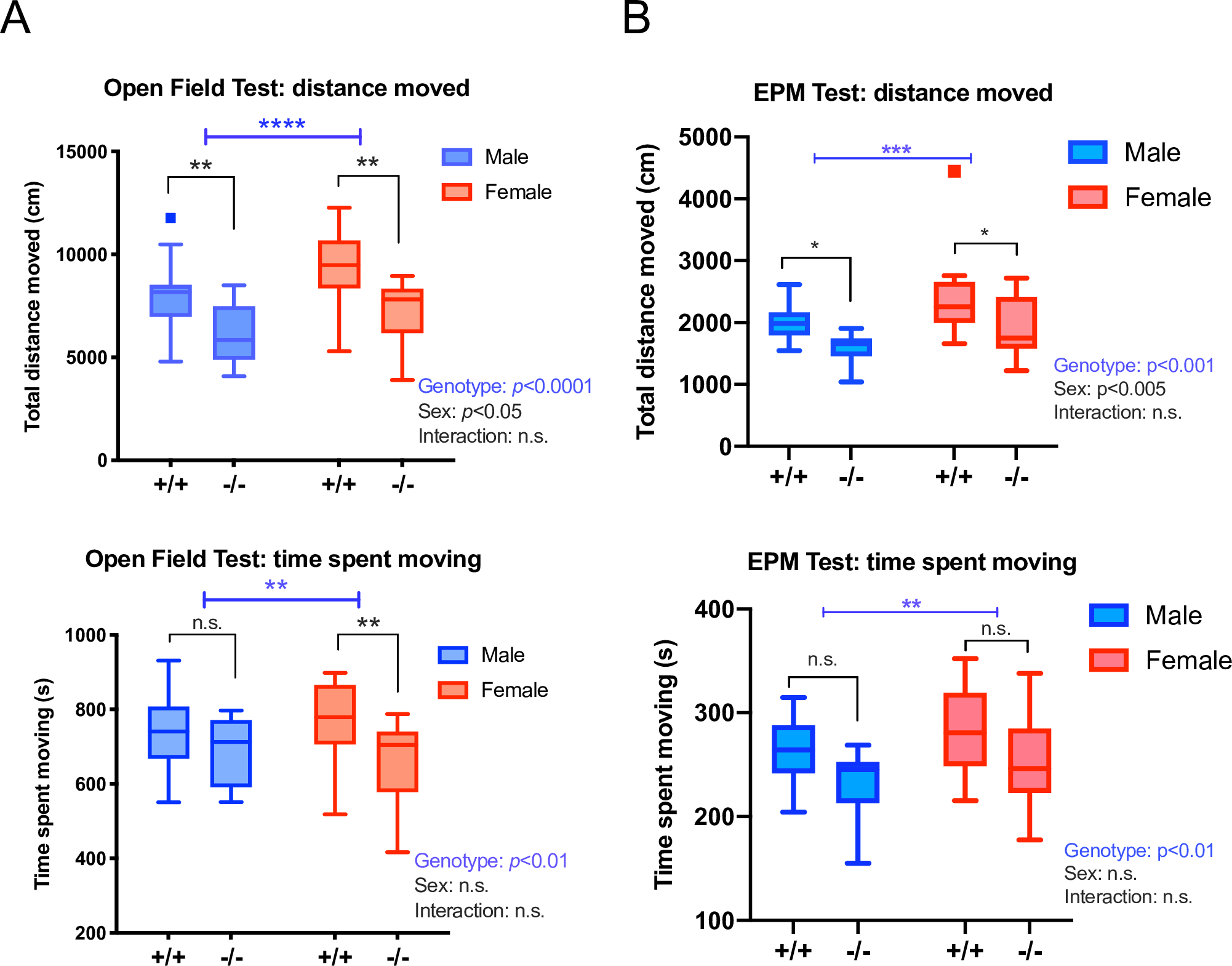

Supplement: S10 Fig — (A) Total distance moved (cm) and time spent moving (s) in the open field. (B) Total distance moved (cm) and time spent moving (s) in the elevated plus maze. Data were analysis by two-way ANOVA (genotype by sex), followed by Sidek’s multiple comparison test between genotypes (+/+ vs. -/-). Male +/+: n = 22, -/-: n = 13; Female +/+: n = 9, -/-: n = 11. “Genotype” factor is highlighted in blue. (TIF) [file pgen.1008916.s011.tif]

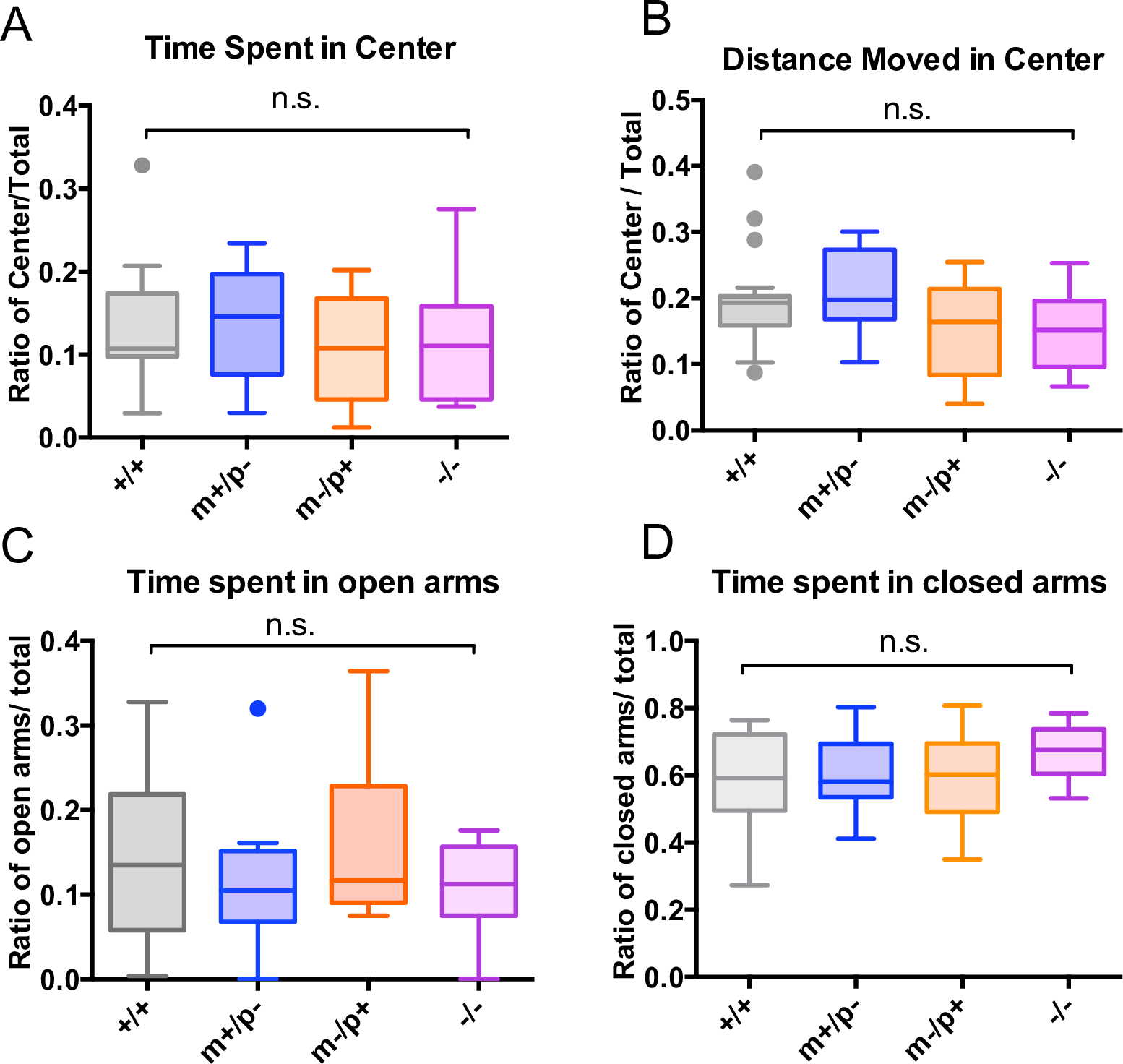

Supplement: S11 Fig — (A) Ratio of time spent in the center zone (total time = 1200s). (B) Ratio of distance moved in the center zone (compared to the total distance moved). (C) Ratio of time spent in the open arms (total time = 600s). (D) Ratio of time spent in the closed arms (total time = 600s). One-way ANOVA with Tukey’s post hoc test. Male mice, n = 22,12,12,13 (+/+, m+/p-, m-/p+ and -/-). (TIF) [file pgen.1008916.s012.tif]

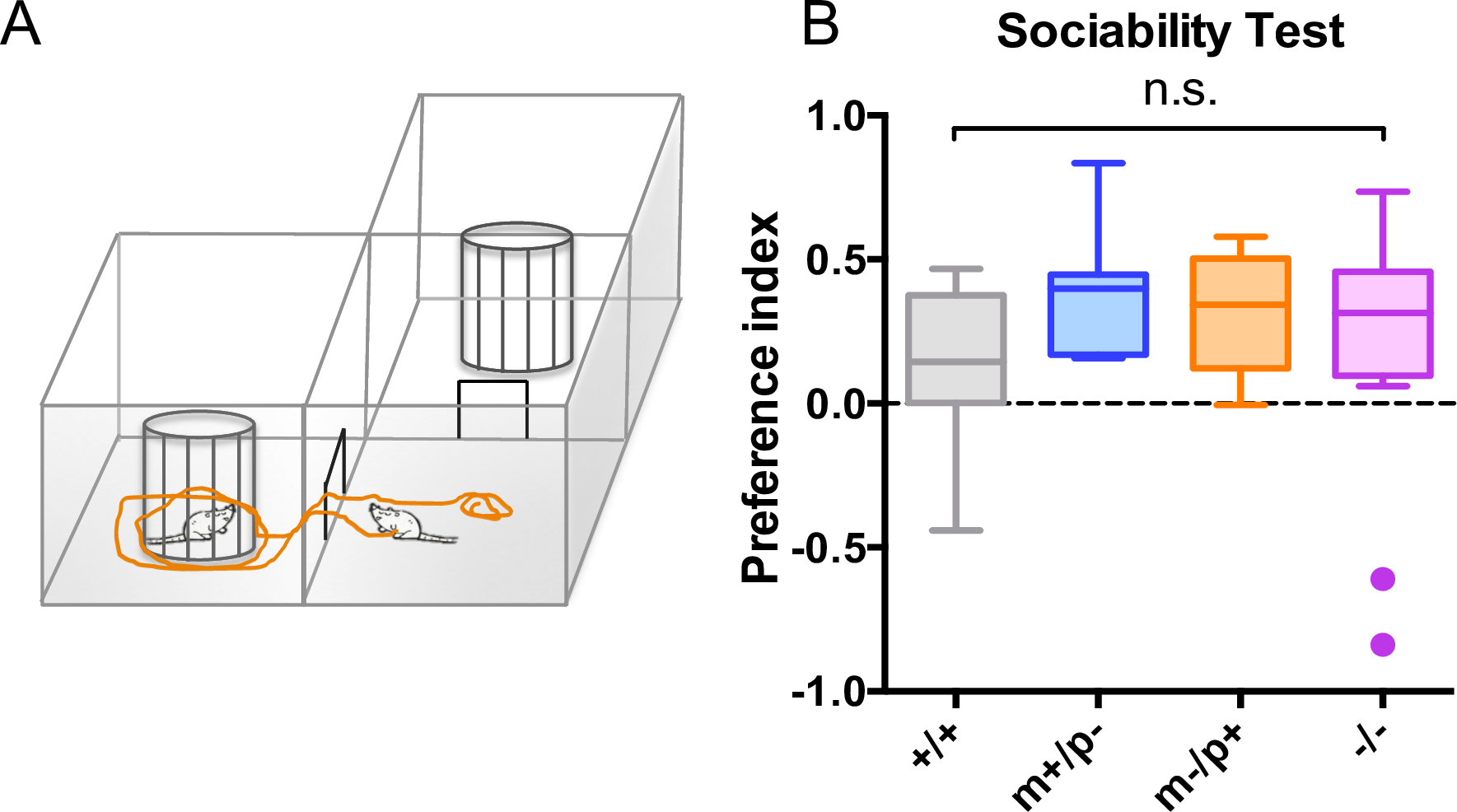

Supplement: S12 Fig — (A) In a “L-shape” three-chamber apparatus, mice were allowed to choose between two chambers: one with a caged live mouse versus another with an empty cage. (B) Time spent in the chamber containing another mouse (“mouse chamber”), chamber with an empty cage (“non-mouse chamber”) and the empty chamber that the test mouse was original released into (“central chamber”) were recorded. Trappc9 homozygous knockout mice (-/-), maternal (m-/p+)/ paternal (m+/p-) heterozygous knockouts and wild-type littermates were compared (males, n = 15,12,11,25 respectively). A preference index (PI) was calculated to score any preference between the two chambers: PI = (duration of stay in the mouse chamber—duration of stay in the non-mouse chamber)/(duration of stay in the mouse chamber + duration of stay in the non-mouse chamber). PI ranges from -1 to 1. One-way ANOVA test with Tukey’s post hoc test: n.s. p>0.05. (TIF) [file pgen.1008916.s013.tif]

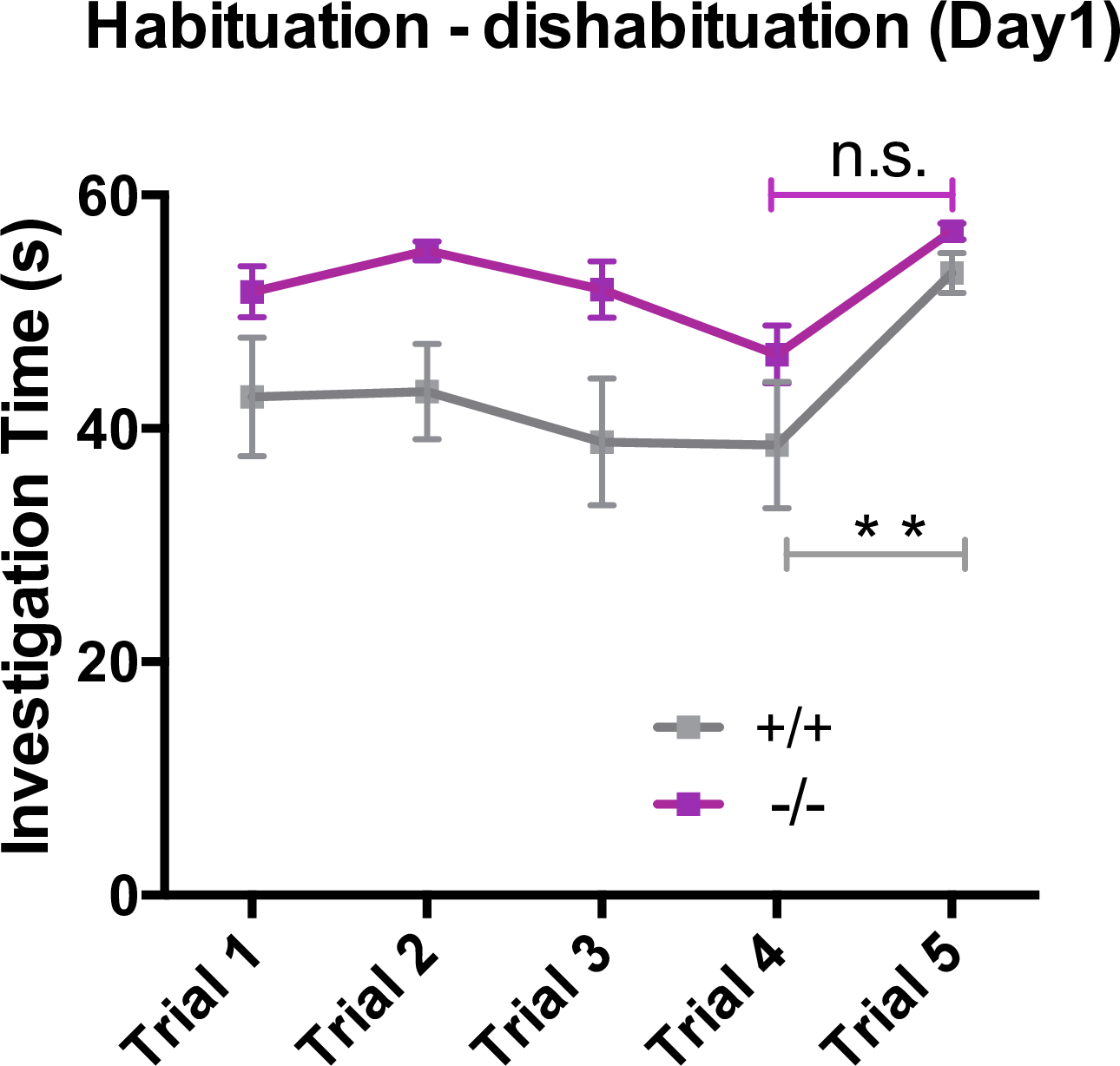

Supplement: S13 Fig — Wild-type male mice showed a significant social learning (grey line, **p<0.01, n (+/+) = 10) at Day 1 of the social recognition test, whereas Trappc9 -/- males (purple line) failed to show such social learning (p>0.05 between Trial 4 and Trial 5, two-tail t-test for 4 and 5 trials, n(-/-) = 9). Trappc9 -/- males also spent significantly longer time to investigate the stimulus mice compared to the wild-types (two-way ANOVA with repeat measurement: p<0.001 for trial and p<0.05 for genotype), may be caused by minimal change of bedding in their home cage, which was applied to reduce male-male aggression. Trappc9 -/- males could not be used for Day 2 test due to lack of social learning at Day 1. (TIF) [file pgen.1008916.s014.tif]

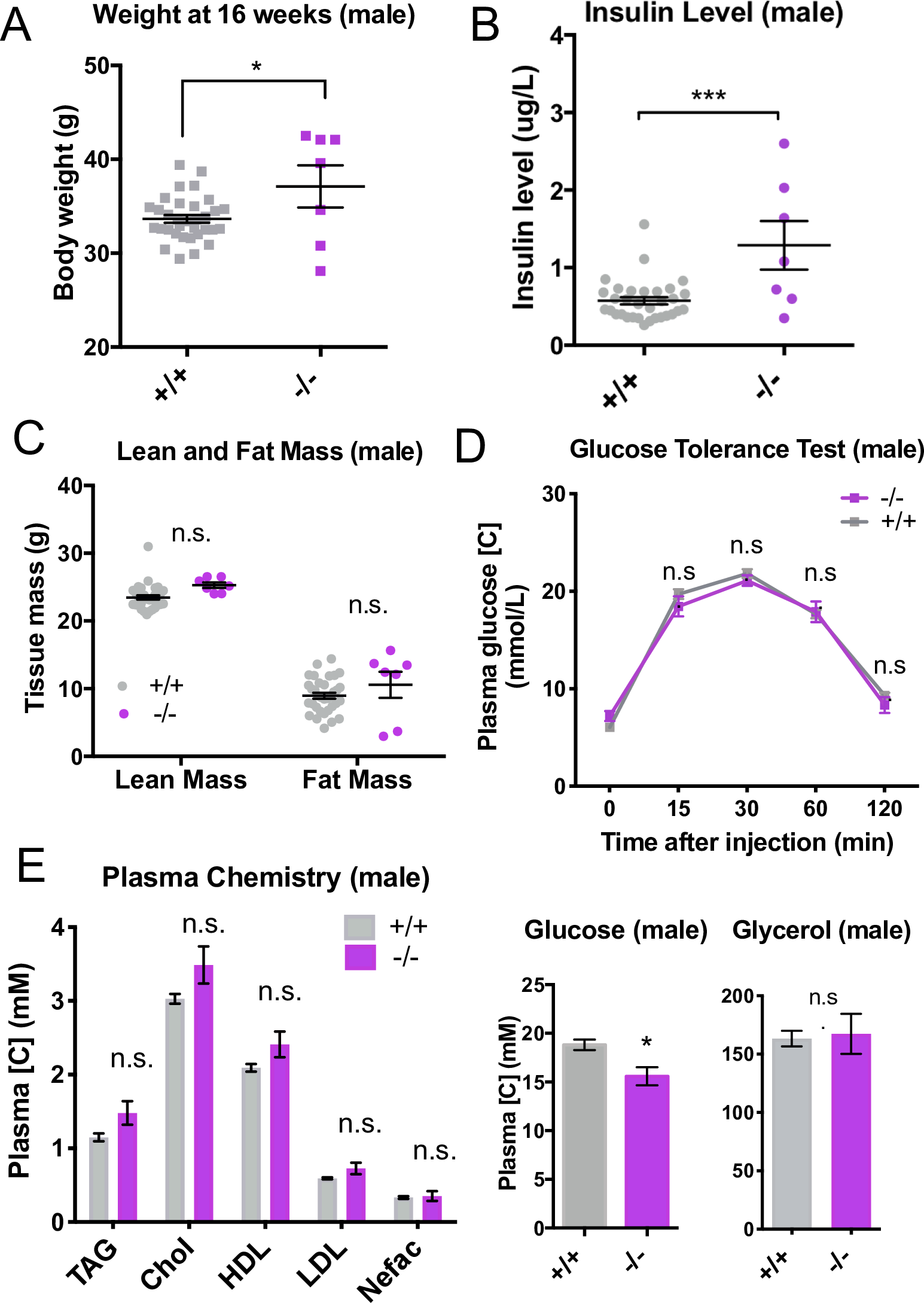

Supplement: S14 Fig — Trappc9 null male mice (Trappc9 -/-, n = 7) showed (A) an increased body weight (p<0.05, two-tailed unpaired t-test) and (B) an elevated blood insulin level (p<0.05, two-tailed Mann-Whitney test) in the phenotyping pipeline analysis, compared to the wild-type controls. This cohort of seven male Trappc9 -/- mice showed normal (C) lean and fat mass, (D) glucose tolerance and (E) levels of triacylglycerol (TAG), cholesterol (Chol), lipoproteins (HDL and LDL), non-essential fatty acid (Nefac) and glycerol in the blood (p>0.05, two-tailed t-test), except for a mild decrease in the blood glucose level (p<0.05). Male wild-type controls: n (+/+) = 32–34, Trappc9 null mice: n(-/-) = 7. n.s. not significant. (TIF) [file pgen.1008916.s015.tif]
